# Design and Experimental Evaluation of a Non-Invasive Microwave Head Imaging System for Intracranial Haemorrhage Detection

**DOI:** 10.1371/journal.pone.0152351

**Published:** 2016-04-13

**Authors:** A. T. Mobashsher, K. S. Bialkowski, A. M. Abbosh, S. Crozier

**Affiliations:** School of ITEE, The University of Queensland, St Lucia, 4072, Brisbane, Australia; University of California San Francisco, UNITED STATES

## Abstract

An intracranial haemorrhage is a life threatening medical emergency, yet only a fraction of the patients receive treatment in time, primarily due to the transport delay in accessing diagnostic equipment in hospitals such as Magnetic Resonance Imaging or Computed Tomography. A mono-static microwave head imaging system that can be carried in an ambulance for the detection and localization of intracranial haemorrhage is presented. The system employs a single ultra-wideband antenna as sensing element to transmit signals in low microwave frequencies towards the head and capture backscattered signals. The compact and low-profile antenna provides stable directional radiation patterns over the operating bandwidth in both near and far-fields. Numerical analysis of the head imaging system with a realistic head model in various situations is performed to realize the scattering mechanism of haemorrhage. A modified delay-and-summation back-projection algorithm, which includes effects of surface waves and a distance-dependent effective permittivity model, is proposed for signal and image post-processing. The efficacy of the automated head imaging system is evaluated using a 3D-printed human head phantom with frequency dispersive dielectric properties including emulated haemorrhages with different sizes located at different depths. Scattered signals are acquired with a compact transceiver in a mono-static circular scanning profile. The reconstructed images demonstrate that the system is capable of detecting haemorrhages as small as 1 cm^3^. While quantitative analyses reveal that the quality of images gradually degrades with the increase of the haemorrhage’s depth due to the reduction of signal penetration inside the head; rigorous statistical analysis suggests that substantial improvement in image quality can be obtained by increasing the data samples collected around the head. The proposed head imaging prototype along with the processing algorithm demonstrates its feasibility for potential use in ambulances as an effective and low cost diagnostic tool to assure timely triaging of intracranial hemorrhage patients.

## Introduction

Traumatic and non-traumatic intracranial haemorrhage (ICH) is a major cause of disability and mortality worldwide [1]. ICH is defined as the extravascular accumulation of blood inside the cranium vault due to the rupture of blood vessels for various traumatic and non-traumatic morbidities, including vascular malfunctions, hypertension and infections [2–4]. The pooling of blood disconnects regular oxygen supply and increases intracranial pressure eventually causing death of the brain and nerve cells permanently damaging the control links with different body parts which can result in a loss of memory, movement or speech and can lead to death. As this devastating disorder deteriorates rapidly, fast diagnosis and management is critically important for the treatment and recovery of the affected patient [5–7].

Advances in neuroimaging techniques have improved the diagnostic capabilities of ICH. To that extent, rapid multimodal CT and MRI are able to identify and locate ICH in acute clinical setting [8–10]. However, the setup of these systems are bulky and require large space, which limits the portability to be carried in an ambulance. Again, these facilities are limited in the rural hospitals due to the cost involved for installation and per scan [11]. In fact, majority of world’s population does not have access to affordable and reliable medical imaging systems [12], especially in low-income countries where healthcare infrastructures are scarce. Numerous researchers are invested in the pursuance of portable and affordable imaging systems. As a result, several portable medical imaging modalities, such as magnetic induction tomography [13], electrical impedance tomography [14], magnetic induction spectroscopy [15], or magnetic induction phase shift spectroscopy [16] are proposed. However, these imaging systems are either invasive or unable to be implemented in realistic environment for ICH detection.

Microwave imaging (MI) is an emerging non-invasive technology that exhibited its potential in breast cancer detection [17–21] and is promising in detecting brain injuries [22, 23]. Although numerical feasibility studies of tomography based MI found the technique promising for imaging a complex environment like the human head, practical test-bed demonstrations are still scarce [24, 25]. While the state-of-the-art MI system [26] demonstrated a valuable milestone towards practical implementation, it requires numerous sophisticated parts and high computational hardware or connection to super-computers which can only be performed in places with well-developed telecommunications systems and hard to achieve in rural areas. Typically, tomography based effective image reconstruction algorithms typically takes long time (in the scale of hours [27]) for image processing, in addition to the data acquisition time (10–15 minutes for breast [17]). On the other hand, wideband radar based MI system offers fast image processing [28, 29], but requires more complex sensing and data acquisition systems than the tomography based MI systems to measure data in a wide bandwidth. Thus automated mono-static scanning systems are seen to take long time for data acquisition (e.g. 30 minutes for breast scan [18]). Arrays provide faster data acquisition, but have strong multipath reflections [19, 20] that eventually reduces the reconstructed image quality by increasing false targets (artefacts). Again, the array based prototypes are difficult to customize for different head sizes due to tens of sensing antennas and are considered bulky [26]. Hence, mono-static scanning is preferred over array based scanning [21]. However, the reported mono-static scanning based prototyped systems suffer from the need for manual scanning [28], which limits the reproducibility of the imaging results.

This paper reports the design and implementation of a non-invasive head imaging system for ICH detection based on wideband microwave imaging technology. The automated, portable mono-static scanning based system is described in detail. An ultra-wideband antenna is designed and prototyped for sensing the scattering fields from an ICH affected human head model. An image theory-supported magnetic symmetry line concept and a cross-feeding technique are applied on a previously reported symmetrical antenna [28] to minimize the space requirements as well as to fix the well-known beam-tilting problem. In order to understand the underlying physics of the head imaging system, head models of different sizes, with different ICH targets in varied locations and excitations are numerically studied. A sophisticated image reconstruction algorithm considering the nature of signal propagation in the complex near-field region is presented. The algorithm takes the surface wave propagation around the head phantom into account and calculates the minimum scattering path on the basis of a permittivity model. The head imaging system is tested on a realistic human head phantom containing frequency dispersive emulated head tissues with MRI-derived anatomical distributions. A stepper motor with a controller unit and a compact microwave transceiver is utilized for fast data acquisition. The reconstructed images demonstrate that the head imaging system with the image processing algorithm is capable to detect and locate the position of ICH brain injuries of various sizes in different depths inside the head phantom. The images are analysed qualitatively and quantitatively using standard metrics, and the results are discussed from different statistical perspectives.

## Head Imaging System

**Fig 1:**
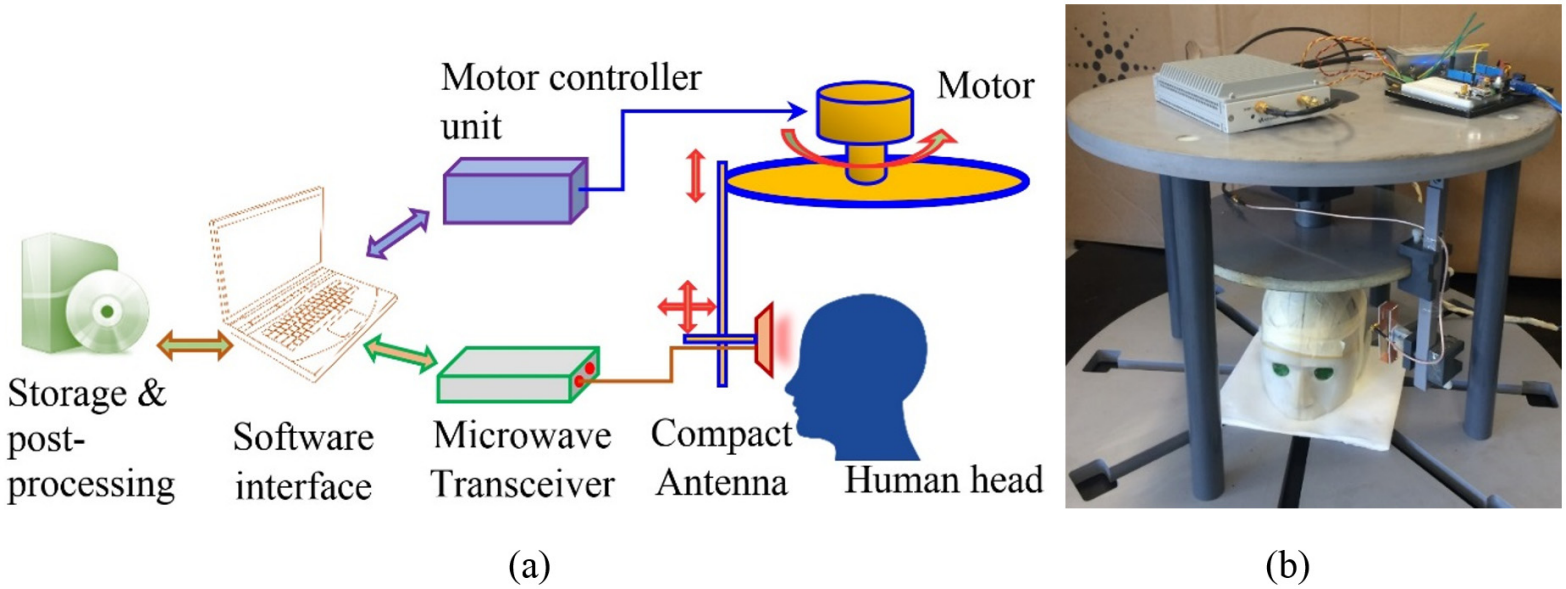
The proposed microwave head imaging system.

The proposed head imaging system is demonstrated in Fig 1. The system can scan the human head by sending low-level electromagnetic fields at microwave frequencies towards the head and measuring the reflected fields from the brain. The system consists of a custom made head imaging platform, a compact wideband antenna, a compact microwave transceiver, a motor controller unit, a software interface, and a storage and post-processing algorithm. The head imaging platform, which is flexible to different head sizes and orientations as per the requirements, holds the DC stepper motor (MYCOM 5-phase DC motor) and other movable system hardware parts. A motor controller unit (MYCOM INS500-120 motor driver) is utilized for controlling the stepper motor, by which the scattered fields are collected from around the head. A compact antenna with wideband operation and directional radiation is designed and prototyped. One sensing antenna is applied in the system to mono-statically transmit and receive electromagnetic signals. The details of the antenna are described later. The antenna is mounted on an adjustable mount. A compact microwave transceiver (model-N7081A) from Keysight, USA is employed for data acquisition. The transceiver is low-cost and light weight with wideband (100 kHz-5 GHz) operating capacity up to a dynamic range of 105 dB [30]. Unlike traditional vector network analysers, which are bulky and expensive, this transceiver enables portability and mass application of the head imaging system. A software interface is designed to control and synchronize the motor controller and microwave transceiver via LAN and USB connections, respectively. The scattering data is recorded and post-processed using a delay-and-sum based signal processing algorithm.

The organization of the rest of the manuscript is as following: the details of the design procedure and characteristics of the wideband antenna is described. Then, the penetration and scattering mechanism of the head scanning system is discussed in order to understand the scattering of ICH targets inside human head in various situations. The radiation safety of the system is checked to ensure that the radiation is below the standard safety limit. Afterwards, the signal processing algorithm is explained in detail followed by the experimental validation procedure and the reconstructed images. The resultant images are quantitatively analysed from multiple perspectives. Finally, the concluding remarks are discussed focussing on the overall performance and advantages of the proposed head imaging system.

### Design and performance of the antenna

The efficacy of microwave-based imaging systems depends strongly on the efficiency of the sensing antennas employed. The antennas capture scattering signals from the imaged body part. Hence, compact antennas with proper impedance matching are essential for a successful imaging system. Additionally, there are several fundamental challenges that need to be considered. The tissues of the head are highly lossy due to their increased conductivity with frequency. Although relatively lower losses and higher penetration depth can be achieved at lower microwave frequencies [24, 31], the reconstructed image using these frequencies suffer from poor resolution. However, operating over a large bandwidth can improve image resolution [32]. Consequently, frequencies from 1–3 GHz are considered a trade-off between these criteria [28]. Nevertheless, the sensing antennas need to be compact and low profile to ensure a light-weight and portable imaging system. Omni-directional antennas appear to be the easiest solutions to meet the system’s requirements. However, since only a tiny fraction of emitted power is scattered back from the head, the near-field characterization of antennas reveal that the directional antennas are more effective for microwave imaging systems [33]. In the near-field region, a unidirectional antenna is able to collect more information from the front side and block more environmental interference from the backward side, increasing the dynamic range of the system [34]. All these requirements increase the complexity of antenna design procedure.

Several antennas are proposed in the literature to meet the aforementioned requirements. Quasi-Yagi, tapered slot or antipodal antennas are commonly referred in wideband microwave imaging [35]. However, these antennas have a large profile along their direction of radiation. Wideband patch or electro-magneto dipole antennas are also proposed as suitable designs with low-profile [36]. Due to the fact that these antennas require large ground planes, they are bulky for imaging systems. Recently, folding and slot-loading technique of three-dimensional antennas are proposed as compact solutions without the need of additional ground plane for directional radiation [28]. The cross-length of the antennas, which mainly limits the number of sensing elements, needs to be miniaturized. Half-cut and feeding modification techniques are therefore proposed [37, 38]. Despite the effectiveness of these techniques, they strongly affect the radiation patterns, making them tilted as a result of high concentration of surface currents on one edge of the antennas [37, 38].

**Fig 2:**
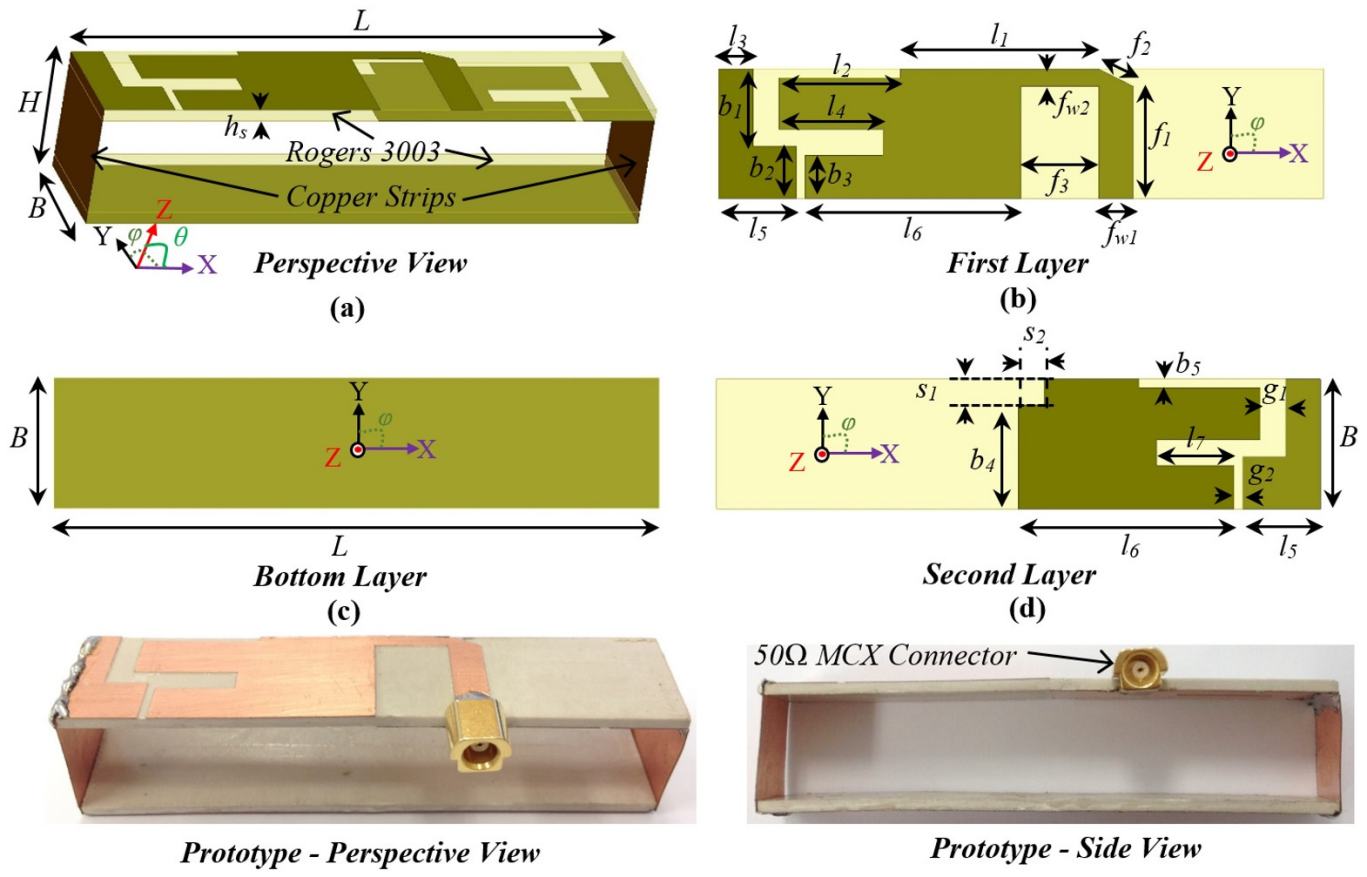
Schematic illustration of the proposed antenna geometry. (a) three-dimensional view, (b) top view of the first printed layer, (c) top view of bottom layer, (d) top view of second layer, (e) perspective view of the fabricated prototype, and (f) side view of prototype.

In order to overcome this radiation deformation limitation of antenna miniaturization, the proposed antenna is designed using a cross feeding technique. Fig 2 illustrates the antenna geometry. Three layers of copper are printed on two slabs of 1.52 *mm* thin Rogers-3003 with permittivity, *ε*_*r*_ = 3 and loss tangent, *tanδ* = 0.003. The antenna is evolved by utilizing the condition of magnetic symmetry along the centre-line of X-axis and transformed to a half-cut structure that has half the area of the original structure [28]. The original structure and thus proposed antenna are based on a slot-loaded three-dimensional folded dipole [39]. This slot-loading technique has the advantage over Yagi-Uda or Quasi-Yagi structure as it attains lower frequencies with smaller size, which is vital for a compact microwave imaging system. The operation of the miniaturized slot-loaded antenna relies on the loop mode for the lowest resonance, whereas the upper frequencies depend on the folded dipole mode. Due to the concentration of strong currents on the top slab, unidirectional radiation patterns are effectively attained. However, it is the feeding structure that makes correction to the radiation beam. As seen from Fig 2(b), the first layer contains the feeding strip, while the second layer is connected to ground. The antenna is fed using a 50Ω microstrip line with feeding width, *f*_*w1*_ = 4 *mm*. Due to the magnetically symmetric half-cut, the real part of the half-cut antenna increases and better matches with higher characteristics impedance. To improve impedance matching, the feeding is terminated with a higher impedance (75 Ω) line having width of *f*_*w2*_ = 2 *mm*, with a truncated transition. The connecting 50Ω microstrip transition line is vital because the system is matched to 50Ω and improper impedance matching induces high reflection and consequently fails to deliver maximum power towards the imaged body. The optimum dimensions of the designed antenna are listed in Table 1. The photographs of the fabricated prototype antenna are depicted in (Fig 1e and 1f). A 50Ω MCX connector is used to connect the antenna to the system. The small structure of the MCX connector assists to lower the inherent radiation from the connector. This is especially important as the antenna is intended to operate in the near-field.

**Table 1:** Geometrical dimensions of the proposed antenna.

**Parameters**	*L*	*H*	*B*	*h*_*s*_	*l*_*1*_	*l*_*2*_	*l*_*3*_	*l*_*4*_	*l*_*5*_	*l*_*6*_	*l*_*7*_	*f*_*w1*_
***Values (mm)***	70	15	15	1.52	23	14	4	12	9	25	9	2
**Parameters**	*b*_*1*_	*b*_*2*_	*b*_*3*_	*b*_*4*_	*s*_*1*_	*s*_*2*_	*g*_*1*_	*g*_*2*_	*f*_*1*_	*f*_*2*_	*f*_*3*_	*f*_*w2*_
***Values (mm)***	9	6	5	12	3	3	3	1	13	4.5	9	4

**Fig 3:**
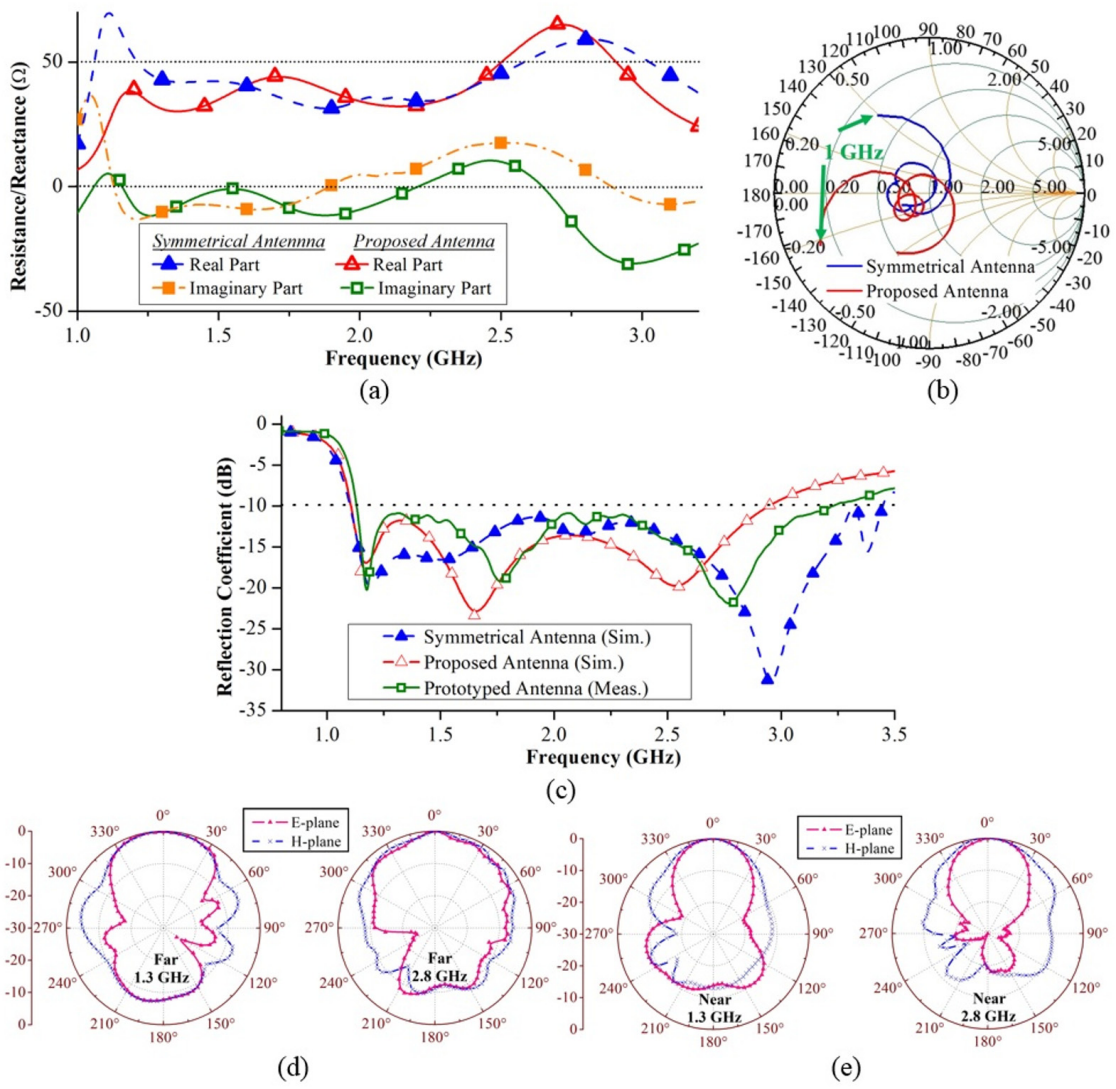
Characterization of the proposed antenna. (a) The impedance comparison over different frequencies with the original symmetrical antenna. (b) Smith chart comparison showing the change of complex input of the antennas. (c) Reflection coefficient results of measured and simulated antennas. The (d) far-field and (e) near-field radiation patterns in E- and H-planes at 1.3 and 2.8 GHz.

Fig 3 shows the impedance transformation of the original symmetrical and proposed half-cut antennas. It is observed in the literature that the symmetrical half-cut yields higher input impedances over wideband that the proposed antenna [38]. From Fig 3(a), it is noted that design of proposed feeding technique effectively reduces the impedance and matches to 50Ω. Nevertheless, the smith chart of Fig 3(b) shows that the complex input impedance mostly relies on the capacitive reactive part and encircles around the impedance locus upon effective feeding implementation. The reflection coefficient plots of the original and proposed antenna is illustrated in Fig 3(c). The proposed antenna attains a wide bandwidth of almost two octaves over 1.1–3.2 GHz. It can be seen that although 50% reduced in size, the optimized half-cut antenna does not sacrifice the penetration capability which is directly related to the lower operating frequency.

The radiation characteristics of the antenna is also measured in an anechoic chamber. Effectiveness of the head imaging system vitally depends on a stable unidirectional radiation pattern towards the desired direction over a wide frequency band. Owing to the high permittivity of the head, the antenna is expected to operate in both far- and near-field regions [37]. The far-field radiation analysis of the antenna shows that the main-beam of the proposed antenna is always towards +Z direction. The cross-feeding technique of the antenna overcomes the main limitation of beam-inclination observed in the previously reported half-cut antennas [37, 38]. As observed from Fig 3(d), both E- (XZ) and H- (YZ) planes achieve unidirectional radiations with a front-to-back ratio (FBR) of around 9 dB which is relatively high considering the electrically small size of the antenna [28]. The near-field radiation is characterized at a 10 cm distance from the antenna surface using a wideband field probe. As shown in Fig 3(e), the antenna attains unidirectional radiation in both E- and H-planes. Due to the high field intensity on the top surface of the antenna compared to the backward reflector in near-field region, the FBR of the antenna is seen to be higher than 12 dB. Furthermore, the narrow beam-width (BW) is noticed in the near-field region. This increased FBR and narrow BW are very helpful for near-field imaging as they significantly reduce multi-path interferences by focusing the beam towards the intended direction.

### Penetration and scattering characteristics of head model

To investigate the radiation characteristics and overall operation of the mono-static imaging system, full wave electromagnetic simulations are performed using CST Microwave Studio, which is a Finite-Integration Technique (FIT) based solver [40]. The antenna is excited using a discrete port representing the SMA-microstrip feeding transition used in practice. An anatomically realistic 3D numerical head model, based on MRI scans of a real patient, is imported for the numerical analysis [41]. The phantom contains 256 × 256 × 128 voxel cubes with each voxel having dimensions of 1.1 × 1.1 × 1.1 *mm*^*3*^. To represent the realistic scenario, each of the seventeen segmented tissues of the 3D phantom are individually assigned with actual measured electrical properties [42]. In the simulations, these broadband dispersive properties can be accurately fitted [43] by the following 4^th^-pole Debye model:

$$\varepsilon_{c}\left(\omega \right)=\varepsilon_{\infty }+\sum_{i=1}^{4}\frac{\Delta \varepsilon_{i}}{1+j\omega \tau_{i}}+\frac{\sigma_{s}}{j\omega \varepsilon_{0}}$$ (1)

Here *ε*_*c*_ is complex relative permittivity as a function of angular frequency (*ω*). The permittivity of free-space (*ε*_0_), relative permittivity at infinity (*ε*_∞_), i^th^ relaxation time (*τ*_*i*_), magnitude of i^th^ dispersion (Δ*ε*_*i*_) and static conductivity (*σ*_*s*_) vary depending on the tissue type within the head. The field distributions in and out of the phantom are analyzed in both time- and frequency domains.

#### A. Time-domain analysis and effect of head size

**Fig 4:**
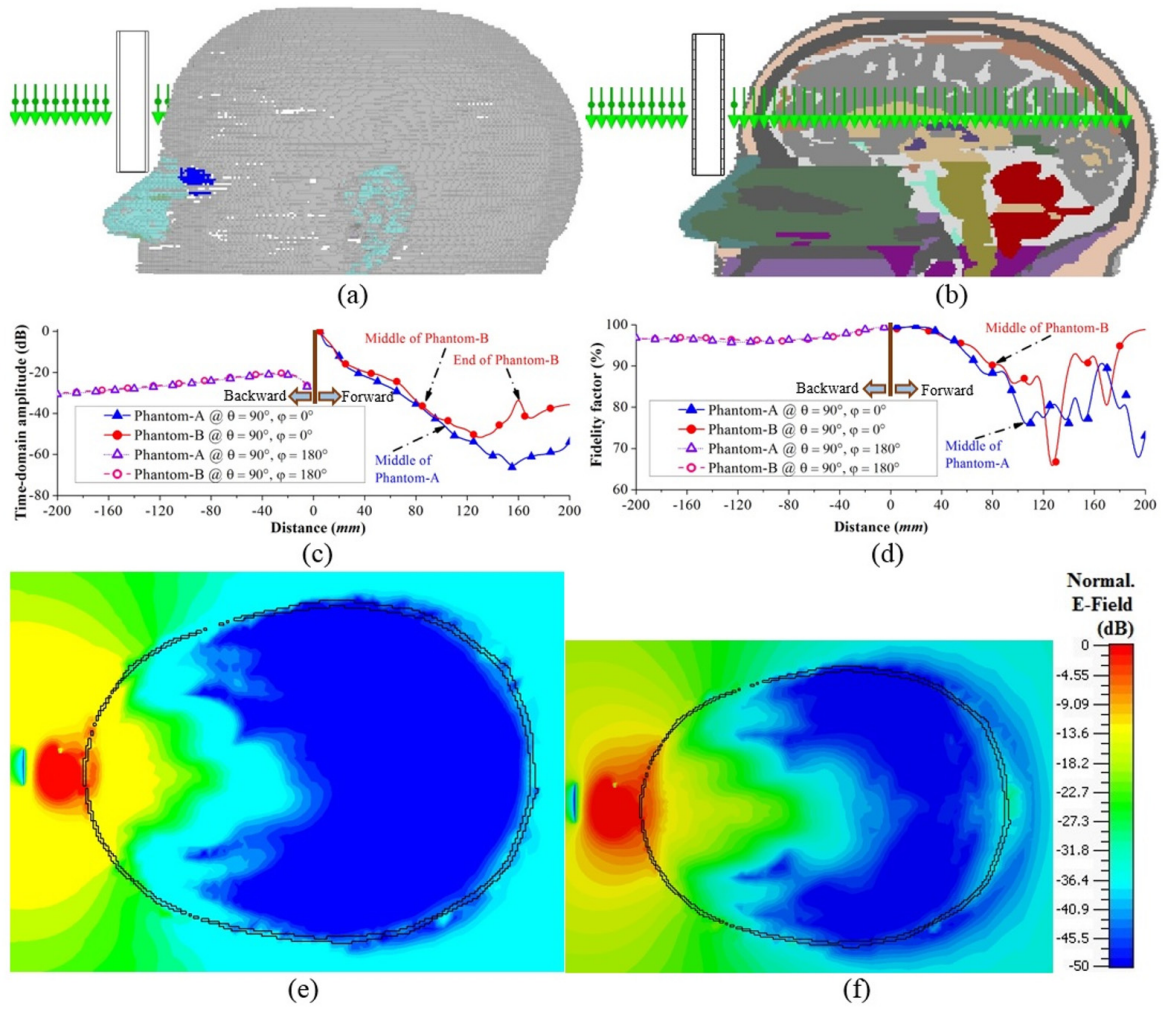
Time-domain analysis of the antenna in presence of different head phantoms. (a) The lateral view of the simulation setup. (b) Midsagittal plane cross-section at the middle of the head phantom showing the positions of E-field probes. (c) The normalized time-domain amplitudes and (d) fidelity factors of the various transient signals at different distances inside and outside the phantoms. Horizontal cross-sections of (e) Phantom-A and (f) Phantom-B, showing the transient E-field distributions normalized to individual maximum.

To analyse the variation of time-domain E-field in the phantom, two arrays of co-polarized E-field probes are placed along the forward and backward directions of the antenna through the mid-line of head phantom. The adjacent probes have a 5 *mm* gap between each other. Fig 4(a) and 4(b) depicts the simulation setup of the numerical study. In order to examine the effects of head size on transient response, a scenario with 25% smaller head phantom (Phantom-B) is also simulated in addition to the original healthy phantom (Phantom-A). The time-domain E-field signals at different probes are measured and their position (*d*) dependent transient amplitudes, *T* and fidelity factors, *FF* are calculated using the following equations [33],

$$T(d)=10log_{10}\left(\frac{Peak\left|E_{d}(t)\right|}{\underset{}{\underset{d}{\text{max}}(Peak\left|E_{d}(t)\right|}}\right)$$ (2)

$$FF\left(d\right)=max\frac{\int_{-\infty }^{+\infty }E_{d}\left(t\right)E_{t}\left(t-\tau \right)dt}{\sqrt{\int_{-\infty }^{+\infty }{\left|E_{d}\left(t\right)\right|}^{2}dt\int_{-\infty }^{+\infty }{\left|E_{t}\left(t\right)\right|}^{2}dt}}$$ (3)

where *E*_*d*_(*t*) is the transient E-field response received by probes in various distances and the template waveform is the generated transient input pulse of choice expressed as:

$$E_{t}\left(t\right)=-sin\left(2\pi f_{c}t\right)exp\left(-2\pi t^{2}/t_{w}{}^{2}\right)$$ (4)

Here, *f*_*c*_ = (*f*_*h*_ + *f*_*l*_)/2 is the central frequency, *f*_*l*_ and *f*_*h*_ are the lowest and highest operating frequencies, respectively. The Gaussian pulse width (*t*_*w*_) is employed so that the pulse spectrum covers the whole operating bandwidth of the antenna in the frequency domain.

Fig 4(c) and 4(d) shows the transient analysis results at various distances inside and outside of the head phantom. Position zero indicates the position of the antenna. It is seen that in close proximity of the antenna, the fields in the front direction are 20 dB stronger than those of the backward direction due to the high FBR of the antenna in the near-field. Moreover, high fidelity factors of around 98% are attained along the front side, which ensures the transmission of distortionless impulses. These characteristics enable the antenna to collect more information from the desired forward side.

In both phantoms (Phantom-A and B) the attenuation slope of the peak E-field amplitude varies as the pulse crosses different tissue types at different distances. Although minor, the field at a depth of 20 to 30 *mm* in phantom-B is seen to be smaller than Phantom-A. This is because in Phantom-B the wave at that distance enters into Cerebrospinal fluid (CSF), Dura and gray matter which have high dielectic properties, while in Phantom-A at that distance, the wave passes fat and skull layers with low electrical properties. As shown in Fig 4(c) and 4(d), values of *T*(*d*) and *FF*(*d*) at the middle of Phantom-A are respectively, -44.47 dB and 80.41%, while, for Phantom-B, the values are -36.38 and 90.49%, respectively. An 8.09 dB enhancement in transient amplitudes and 10.08% improvement in fidelity factors are attained by 25% reduction of the head size. This conclusion is significant because it reveals how the detection capability, which is related to the signal attenuation inside the head, of a mono-static head imaging system varies from one head to another. It is expected that the size and tissue distribution of the head vary from one patient to another.

The normalized transient field distributions inside the head phantoms at the mid-cross section of the antenna are illustrated in Fig 4(e) and 4(f). In contrast to Fig 4(c), the complete map of head’s cross-section including the discontinuities of *T*(*d*) at the skin boundaries incepted by the reflections can be observed from the heat-map of Fig 4(e) and 4(f). However, it can be seen from Fig 4(e) and 4(f) that the discontinuities of the transient fields at the boundary are not continuous, as the head phantoms include inhomogeneous layers of skin. Again, owing to its smaller size of Phantom-B, higher transient E-field distributions are observed in as compared to Phantom-A. However, in the smaller phantom-B, stronger surface waves are also observed. The surface waves yield multi-path interference, which is added to the direct waves travelling through the phantom. The effect of multi-path waves can also be observed in Fig 4(c) and 4(d). It is seen that the fidelity factors rapidly fluctuate after the middle of the phantoms because there is no longer a direct travelling wave, but summation of multiple waves. For the same reason, the transient amplitude attenuation slope gradually reduces and then increases. However, the minimum path calculation can be used to mitigate this multi-path interference as described in the image processing algorithm section.

#### B. Analysis of broadband frequency-domain scattering characteristics

Although the time-domain field distributions illustrate the overall radiation scenario inside a healthy head phantom, frequency-domain characterization is also important to understand the scattering behaviour of hemorrhagic targets inside the head. The electromagnetic scattering of a target from a non-magnetic, dielectrically heterogeneous domain at a measurement location of ***r***, can be reliably modelled [44] by the following volume integral equation:

$$\Delta E_{s}(r)=\omega^{2}\mu \int_{\Omega }\overset{}{\overset{=}{G^{b}}(r,r\prime )E^{t}(r\prime )[\varepsilon^{t}(r\prime )-\varepsilon^{b}(r\prime )]dr\prime }$$ (5)

where Δ***E***_*s*_ is the total scattered field representing the total observed field difference between the target environment and the background environment. Ω is the investigated region. The Green’s dyadic function ${\bar{\bar{G}}}^{b}$ provides the field inside the head without the target. The relative permittivity of the target and background are denoted by *ε*^*t*^ and *ε*^*b*^ accordingly. ***E***^*t*^ is the total incident electric field due to the source antenna in the unhealthy head environment. The target has the same the relative permittivity of blood, which spreads from 61–57 over the band of 1.1–3.2 GHz. The background in the imaged domain varies due to the inhomogeneous distribution of head tissues and the probable diverse positions of the hemorrhagic target inside the head. Nevertheless, in order to understand the complex scenario and contrast of the target, properties of the possible background tissues are worth to mention. The relative permittivity values of grey matter, white matter and CSF over the operating band are 52–48, 39–36 and 68–64, respectively [42]. The target typically does not form only in one type of tissue, rather it is situated in a mix of the aforementioned tissues and many others. Hence, in order to comprehend the inhomogeneous scenario, the 3D FDTD simulation is performed in CST environment and the total scattered field of the hemorrhagic target is sequentially post-processed by subtracting the healthy case from the ICH affected case. Various aspects of scattering of hemorrhagic targets are considered.

**Fig 5:**
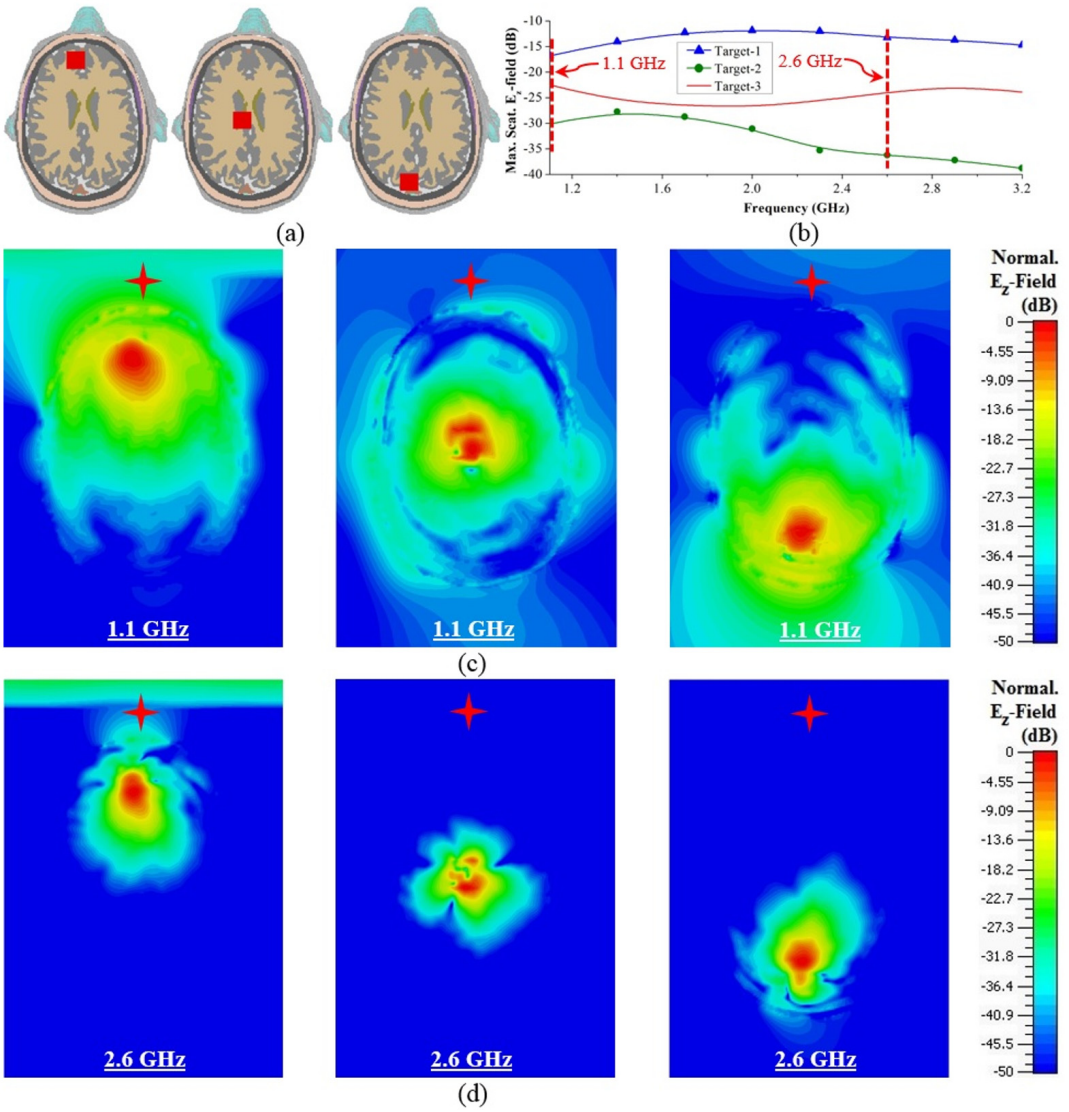
Frequency-domain analysis of ICH affected head with different target positions. (a) Cross-sectional view of the realistic head showing the positions of the ICH targets in front (target-1), middle (target-2) and back (target-3) positions. (b) Maximum E_z_-field scattered by different targets over the utilized band of operation. Horizontal cross-section illustrating the distributions of the scattered E_z_-field normalized with respect to their individual maximum E_z_-fields at (c) 1.1 GHz and (d) 2.6 GHz as marked in (b). Red stars indicate the positions of the antennas.

**(i) Hemorrhagic scattering in different depths:** ICH can occur at any point inside the brain parenchyma [1–3]. Hence, it is important to understand the scattering of haemorrhage at different depths inside the head. Fig 5(a) exhibits the cross sections of Phantom-A having hemorrhagic targets of 2×2×1 cm^3^ in front (target-1), middle (target-2) and back (target-3) positions. While the head phantom is illuminated with the antenna from θ = 90° and φ = 0°, different frequency component of the operating bandwidth yields different scattering output depending on the position of the scatterer. The peak value of the relevant co-polarized components, E_z_ of Δ***E***_*s*_ in different frequencies are calculated and normalized to the impinging electric field on the head phantom, which is 1 V/m. As illustrated in Fig 5(b), the higher frequencies in most cases suffer more losses due to the reduction of signal penetration. Again depending on the position of the targets, the maximum scattering of E_z_-field varies. It is seen that the in shallow regions, both target-1 and -3 scatters more strongly than the deep target-2. However, target-1 emits stronger fields than target-2. This is because the former is closer to the excitation and scatters the received direct travelling fields, while the latter is far from the source and mostly interacts with the surface waves rather than the direct travelling waves.

The cross-sections of the head model showing the scattered E_z_-field distributions for 1.1 and 2.6 GHz are illustrated in Fig 5(c) and 5(d) for the three targets. The images are normalized with respect to the maximum E_z_-field of each frequency plotted in Fig 5(b). It can be seen that while the source is at θ = 90° and φ = 0°, all three targets can scatter back to the source within -40 dB at 1.1 GHz with the help of direct scattering waves or scattering surface waves. However, at 2.6 GHz, the targets far from the antenna are unable to strongly scatter back to the antenna, especially for the deep target-2 because of the high losses related to the high frequencies. Hence, lower frequencies are more effective for target detection.

**Fig 6:**
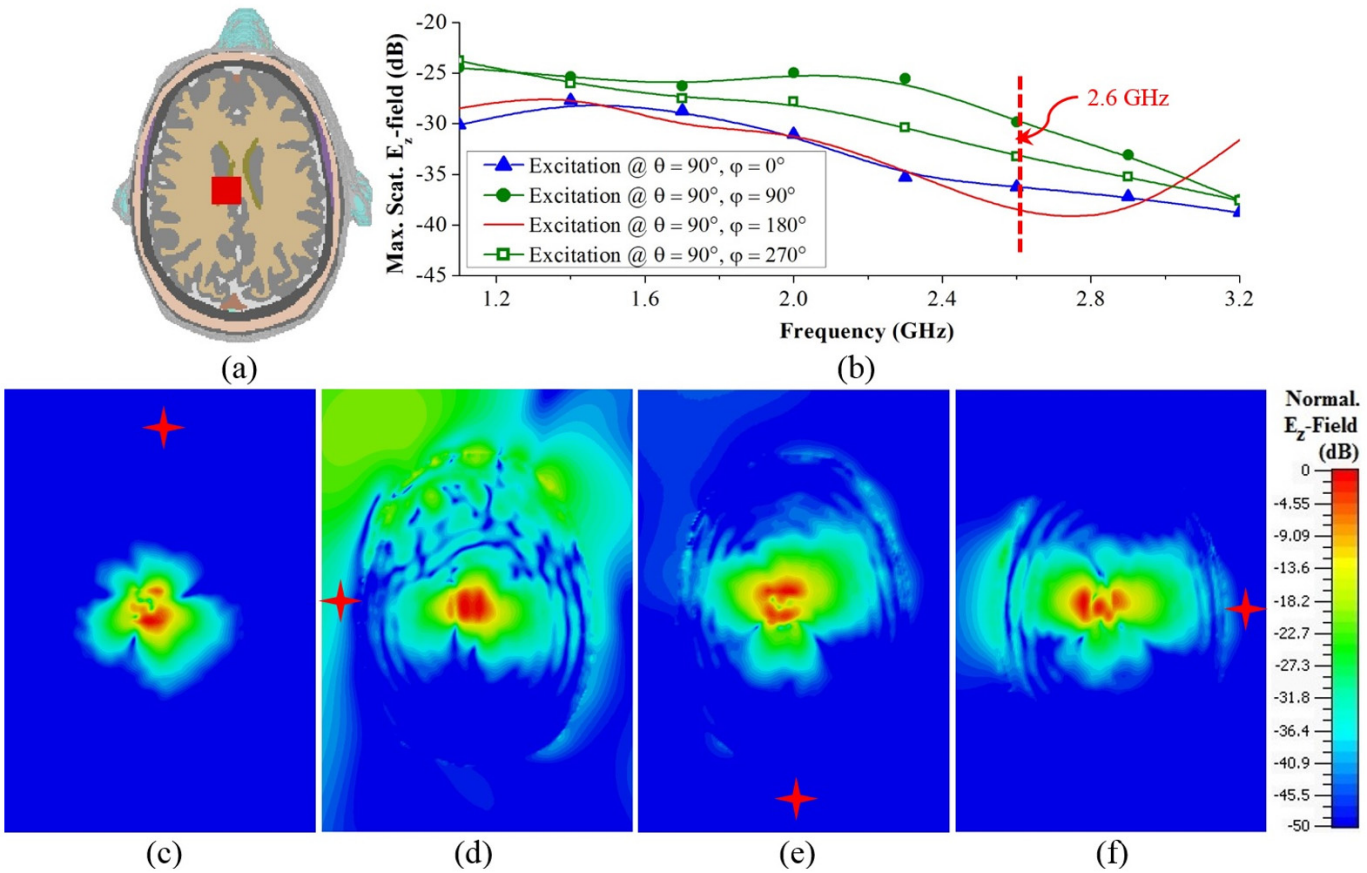
Frequency-domain analysis of ICH affected head with deep target-2. (a) Horizontal cross-sectional view of the human head model. (b) Maximum scattered E_z_-fields over the operating band when the excitations are placed in different positions around the head. (c) Cross-sectional view of the scattered E_z_-field distributions at 2.6 GHz, normalized to individual maximums as indicated in (b). Red stars represents the positions of the excitations.

**(ii) Hemorrhagic scattering with source at different direction:** As shown in Fig 5, at high frequencies, the scattered fields from deep targets are very weak and hence are difficult to detect. However, using only lower frequencies results in poor resolutions of the reconstructed images. Thus, the phantom with target-2 is illuminated from different directions and peak E_z_-fields are calculated and normalized to the maximum radiated fields (Fig 6(a) and 6(b)). Since the target is at similar distance away from the front (φ = 0°) and back (φ = 90°) positioned antennas, the maximum scattered E_z_-field values are quite comparable. But, when the antennas are placed at the sides, the distance for the signal to travel to the target is smaller. Consequently, the emitted E_z_-fields are also higher. However, as the left-sided (φ = 90°) antenna is closer to the target than the right-sided (φ = 270°) one, it scatters more. Fig 6(c) and 6(f) depicts the normalized E_z_-field distributions in the cross-section of the head model with different source positions at 2.6 GHz. Although the target is unable to scatter back to the source within -50 dB for excitations at φ = 0° and φ = 90°, it is effective for the excitations at φ = 180° and φ = 270°. Thus, at higher frequencies, even though the deep targets might not scatter strongly for longer radii of the head phantom, they are detectable for antennas facing smaller radii.

**Fig 7:**
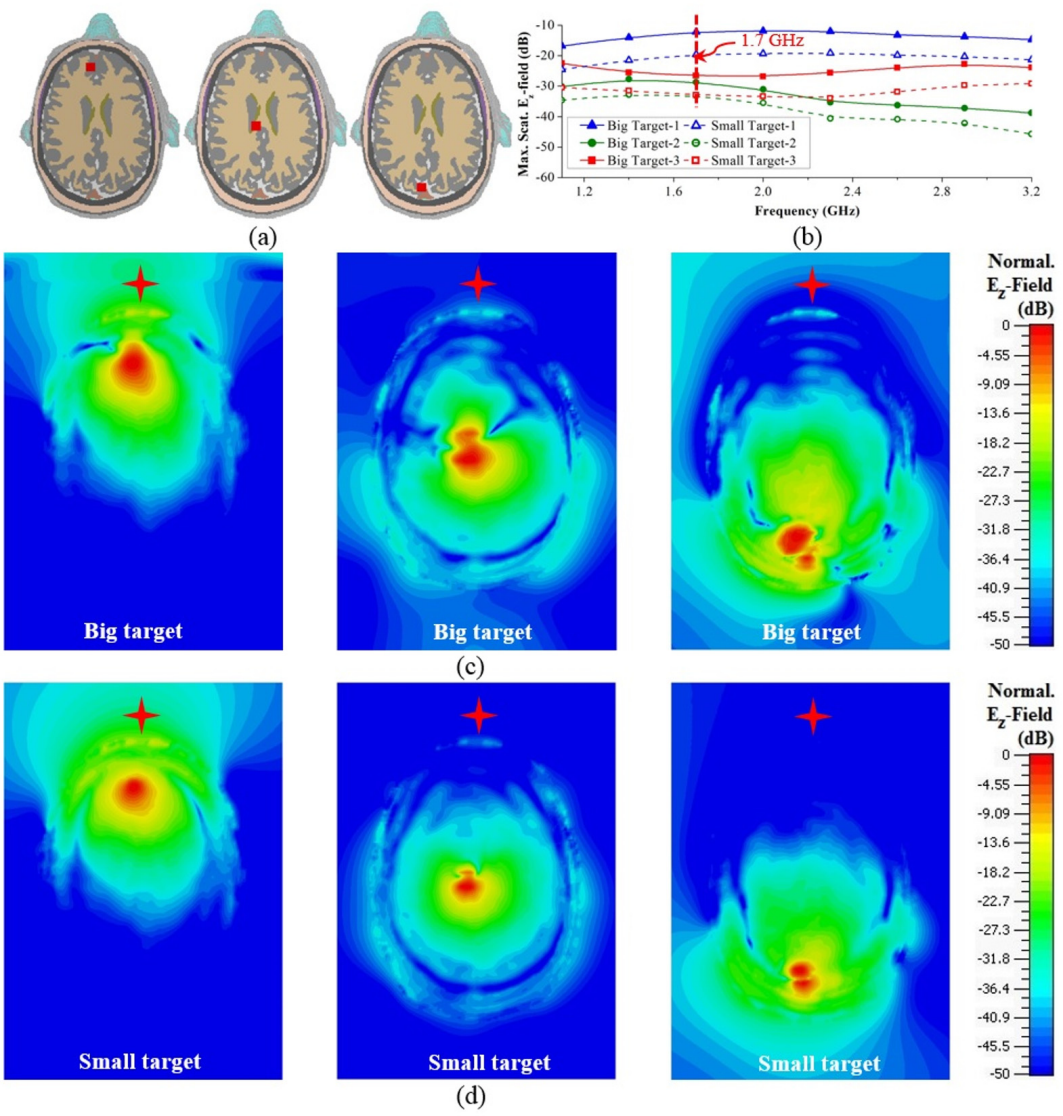
Frequency-domain analysis of ICH affected head with different target sizes. (a) Head cross-sections depicting the positions of the small targets. (b) Comparisons between the peak E_z_-fields scattered by the big and small targets over the band of operation. (c) Horizontal cross-sections showing the E_z_-field distributions that are normalized with respect to the peak values pointed in (b). The locations of the excitation sources are indicated by red stars.

**(iii) Hemorrhagic scattering of different target sizes:** To analyse the effects of target size reduction, another set of simulations is performed with 1×1×1 cm^3^ target at the same positions and with the same source position mentioned in Fig 5. The normalized maximum scattered E_z_-field at different frequencies due to the smaller target at different positions are recorded. As seen from Fig 7(b), 8–10 dB reduction in the maximum scattered E_z_-fields is obtained due to the reduction of target size. The cross-sectional E_z_-field distributions, normalized to the individual maximum values are demonstrated for 1.7 GHz in Fig 7(c) and 7(d). It is observed that although the shallow targets are still able to send strong scattered signals, the deep target-2 suffers from low scattering. Increasing of the dynamic range of the microwave transceiver may solve this issue.

### Radiation safety of the system

**Fig 8:**
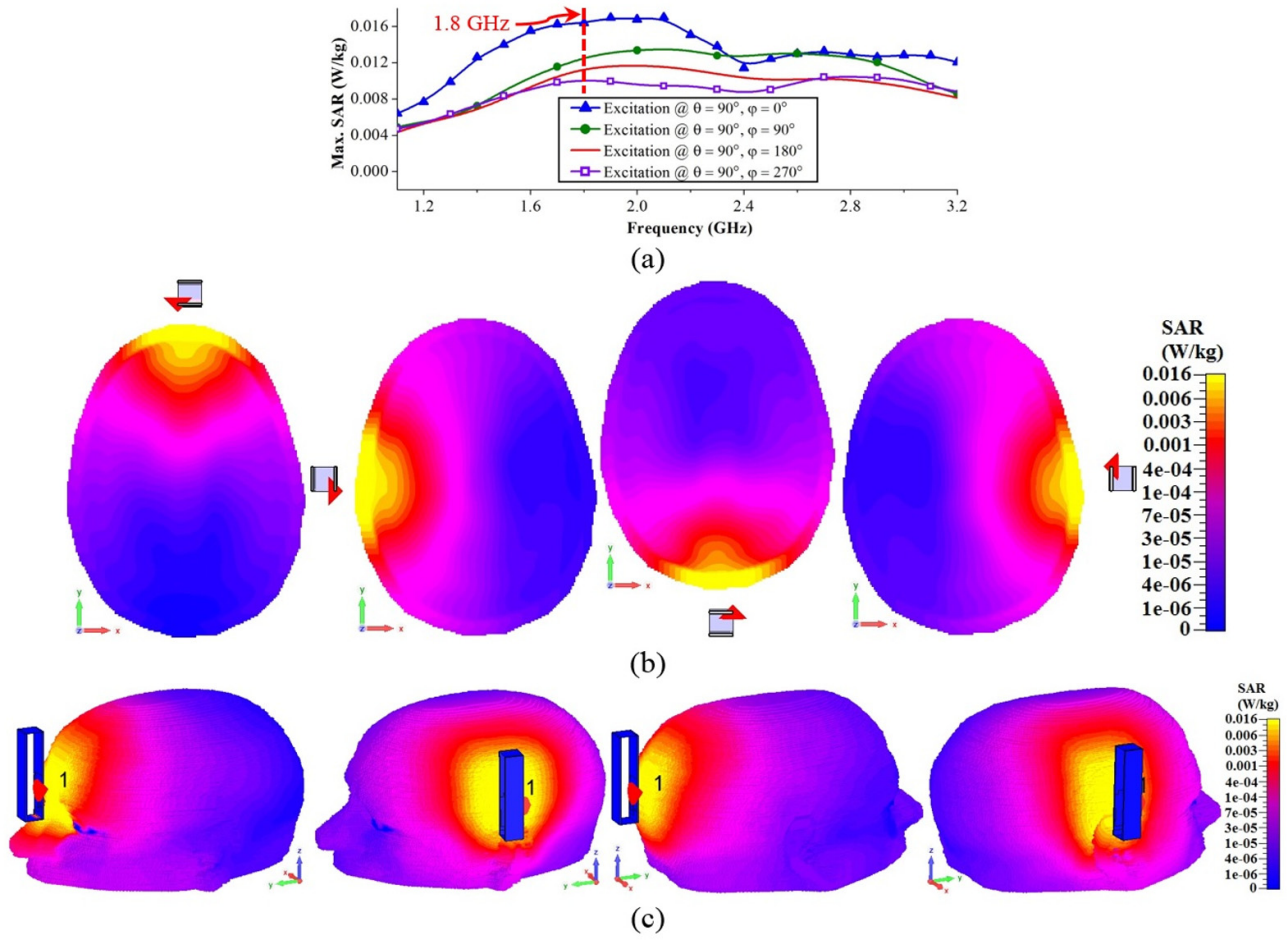
Results from SAR calculations to analyse radiation safety of the imaging system. (a) Maximum SAR values with respect to different frequencies and different antenna positions. (b) Cross-sectional and (c) 3D-view of SAR distributions of the realistic head model with different source locations at 1.8 GHz. The colour bar represents SAR values at 1.8 GHz; yellow colour represents the highest SAR, while blue is the lowest.

Radiation safety is a concern for the implementation of the head imaging system. To that end, the specific absorption ratio (SAR) inside the head is calculated for the mono-static imaging system. In 3D simulation environment, the MRI derived realistic head model is illuminated with the antenna from various directions. The system operates with a transmission power of 0 dBm or 1 mW. The maximum SAR values at different frequencies and for various source locations are presented in Fig 8(a). It is observed that when the source is located in the front side (θ = 90° and φ = 0°), the SAR values are highest compared to the other source locations. At low frequencies, SAR is the lowest and gradually increases with fluctuations. This suggests that the losses are low at low frequencies, but are frequency dependent. All the peak SAR values are lower than 2 W/kg, however, which satisfies the IEEE radiation safety limit [45] for microwave devices. The cross-sectional and perspective view of SAR distributions at 1.8 GHz are depicted in Fig 8(b) and 8(c). It is noted that the maximum SAR is located on the skin layer of the head indicating the lossiest part for the microwave signals. The SAR distributions reveal that SAR values gradually become smaller with the increased tissue depth.

### Signal processing and imaging algorithm

**Fig 9:**
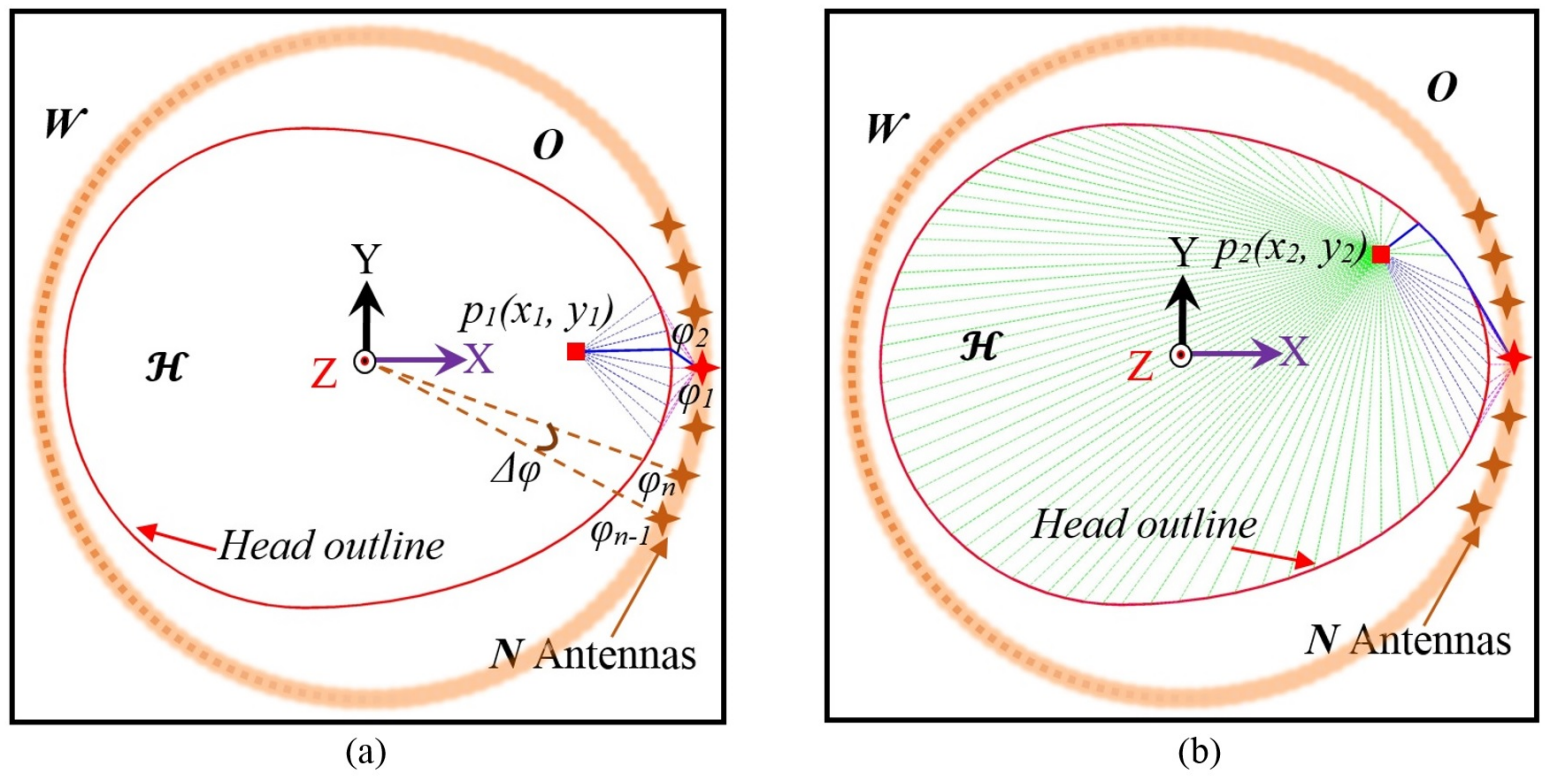
Illustration of data acquisition from different angular locations around head and minimum path calculations for different points inside head from antenna-1 perspective.

The data acquisition system utilizes a circular scanning approach, symmetrical in the mid-sagittal plane (Fig 9). It measures N different antennas where each angular position *n* represents a position within the range from 1 to *N*, and adjacent antenna’s positions have angular differences of Δ*φ* = 360°/*N*. A signal is sent from the compact transceiver through the antenna, which illuminates the head. The reflected signal is measured via the same antenna thereby using a monostatic scanning approach.

The transceiver converts the received signals to *M = 284* frequency samples covering the band 1.1–3.2 GHz. These samples are the complex frequency–domain reflection coefficient, ***Γ***(*φ*_*n*_, *f*_*m*_) where *m* = 1 to *M*. and *n* = 1 to *N*. In order to apply the radar-based imaging approach, the acquired frequency domain signals (***Γ***(*φ*_*n*_, *f*_*m*_)) are converted to time domain (***T***(*φ*_*n*_, *t*_*k*_)) for each antenna through inverse discrete Fourier transformation.

$$T\left(\varphi_{n},t_{k}\right)=\text{exp}\left\{\left[S_{k\times m}\right]\right\}\times \Gamma \left(\varphi_{n},f_{m}\right)=\left[\begin{array}{ccc} \text{T}\left(\varphi_{1},t_{1}\right) & \cdots & \text{T}\left(\varphi_{N},t_{1}\right)\\ \vdots & \ddots & \vdots \\ \text{T}\left(\varphi_{1},t_{m}\right) & \cdots & \text{T}\left(\varphi_{N},t_{k}\right) \end{array}\right]$$ (6)

where $\Gamma \left(\varphi_{n},f_{m}\right)=\left[\begin{array}{ccc} \Gamma \left(\varphi_{1},f_{1}\right) & \cdots & \Gamma \left(\varphi_{N},f_{1}\right)\\ \vdots & \ddots & \vdots \\ \Gamma \left(\varphi_{1},f_{M}\right) & \cdots & \Gamma \left(\varphi_{N},f_{M}\right) \end{array}\right],$

$$f_{m}=f_{l}+\left(m-1\right)\left(f_{h}-f_{l}\right)/\left(M-1\right)\;\text{and }\left[S_{k\times m}\right]=\left[\begin{array}{ccc} \text{j}\omega_{1}t_{1} & \cdots & \text{j}\omega_{m}t_{1}\\ \vdots & \ddots & \vdots \\ \text{j}\omega_{1}t_{m} & \cdots & \text{j}\omega_{m}t_{k} \end{array}\right]$$ (7)

Where *c* states the speed of light in free space, *ω*_*m*_ = 2*πf*_*m*_ represents the angular velocities of different frequencies in free space and *k* is number of equidistant points starting from the antenna’s radiation point to the distance where the amplitude of time-domain signal is negligible. Eq (6) considers the positive distances from the radiation point located in free space.

Signals transmitted from the antennas faces substantial reflections from the air-skin interface. It is noted that a healthy human head is largely symmetric across the mid-sagittal plane [46, 47]. The human brain is often described as pairs of left and right-hemisphere homologues [48] and the relative symmetry is reported to be utilized as baseline for detecting tumors [49]. In order to acquire the effective scattered signals from the head interior, it is assumed that since the antenna positions are located symmetrically around the head and because of the relative symmetric distributions of the head, the antenna at symmetrical positions face similar reflections from the air-skin interface. Thus, the scattered signals from head interior are computed by subtracting the average signal of symmetrical antenna locations facing the right and left-hemispheres using the following equation

$$D\left(\varphi_{n},t_{k}\right)=T\left(\varphi_{n},t_{k}\right)-woody\;avg\left(T\left(\varphi_{n},t_{k}\right),\;T\left(\varphi_{N+2-n},t_{k}\right)\right)$$ (8)

Here, the Woody average representing the average with first alignment of individual signal, is employed which is also proven to be effective for biomedical and imaging applications [50]. This condition of symmetry might fail if the target is in symmetry to all antenna locations and thus there is a possibility of false negative detection. However, it is highly unlikely situation as ICH typically occurs in either the right or left hemisphere [48].

In order to form an image, an imaging area of 0.3 × 0.3 *m*^*2*^ is divided into P×Q cells, with a fine resolution of 0.5×0.5 *mm*^*2*^, where cells are defined by their individual center points at (*x*_*p*_, *y*_*q*_) where *p* = 1 to P and *q* = 1 to Q. A boundary vector, ***B***_***i***_, representing the skin-air interface in Euclidean space, is defined as the outline of human head. Here, ***i*** = 1 to *I*, states the number of considered points on head outline. Wave propagation from the phase center of the antenna to a suspected point inside the head consists: *1)* a path from the excitation point to the skin-air interface outside the head, ***O*** and *2)* a route inside the head H, from head outline to the suspected point. According to Fermat's principle of least time, the wave propagates along the shortest possible electrical path *L*_*min*_ (*x*_*p*_, *y*_*q*_) and thus the propagation time index ***τ*** can be calculated as,

τ(xp, yq)= τant+Lmin (xp, yq)c (9)

The time required by the antenna to radiate electromagnetic waves from the inception of the excitation is defined by antenna delay, *τ*_*ant*_. All possible paths for wave propagations, ***L***(*x*_*p*_, *y*_*q*_) from an antenna to a point (*x*_*p*_, *y*_*q*_) inside the head can be defined as,

$$L\left(x_{p},\;y_{q}\right)=\;\|\varphi_{n}-B{}_{i}\|+\sqrt{\varepsilon_{eff}}\|B_{i}-p\|\;\text{where }p\;\epsilon \;H$$ (10)

Here, the effective head permittivity, *ε*_*eff*_ is a constant in previously utilized algorithms [28]. This assumption results in inaccurate calculations which lead to errors in image reconstruction. This is because the heterogeneous distributions of the head tissues have a diverse values of electrical properties and hence, using a homogeneous permittivity for all of the tissue types gives inexact computations and results in artefacts in the reconstructed images. A model for the effective dielectric constant of the human head, considering point of entry of the signal and distance of the suspect point inside the head, is implemented in the current image processing algorithm.

As the radiated signal enters into head, it has to first interact with an irregularly thin high permittivity skin layer and then it penetrates through thick fat and skull layers, which have low permittivity. Afterwards, the signal goes through very high permittivity CSF and Dura tissues, and gradually passes through grey and white matters, which have high permittivity. To that end, a realistic simulation model is studied in CST Microwave Studio environment with the previously discussed MRI-scan derived healthy human head and the effective permittivity values of the healthy human head for different ***φ***_***n***_ antenna positions are calculated over the band 1.1–3.2 GHz by analysing the differential time of signal arrival (TOSA) from the point of entry with the procedure described in [29]. It is realized from simulations and from [29] that the effective permittivity of a point inside the head is different for different entry points of the head boundary and depends on the distance of that arbitrary point from the boundary. In order to deduce a unified propagation model, the values for different point of entry are averaged and the result is expressed using a distance based function for any entry point of the head boundary as,

$$\varepsilon_{eff,\;i}=\varepsilon_{max}\left(1-\alpha e^{-\beta d/a}\right)$$ (11)

Here the distance of the arbitrary point from the boundary is represented by *d*, the normal distance from the signal entry point to the center of the head is *a*, maximum permittivity is defined by *ε*_*max*_, and *α* and *β* are constants. For the band of 1.1–3.2 GHz, the effective permittivity values from the realistic simulation environment suggests values of *ε*_*max*_ = 41, *α* = 0.75 and *β* = 6.4.

In a mono-static radar approach, the transmitted signal travels to and is scattered from the arbitrary point (*x*_*p*_, *y*_*q*_); thus, the total propagation time is proportional to twice the distance

$$\tau_{total}\left(x_{p},\;y_{q}\right)=2\tau \left(x_{p},\;y_{q}\right)$$ (12)

It is understood that the line of sight points (***B***_***i***__,__***n***_) receive direct signals from the antennas and the waves propagate via these points through head interior. These points are the tangent points from H from individual antenna position and the points between them. The tangent points are computed by estimating the maximum angle, *θ*_*max*_ centered at antenna position from $B_{i_{max},\;n}$ to $B_{i_{min},\;n}$. Thus, Eq (10) can be written as,

$$\begin{array}{l} L\left(x_{p},\;y_{q}\right)=\;\|\varphi_{n}-B_{i,n}\|+\sqrt{\varepsilon_{eff,i}}\|B_{i,n}-p\| \end{array}$$ (13)

It is observed that Eq (13) is effective for propagation path estimation of deep points inside the head which usually follow the line of sight paths due to minimum losses (Fig 9(a)). If the target appears deep from the sensing antenna and is shallow from another angle, it is likely that strongest scattering signals will reach the skin boundary following the shortest path and then propagate as travelling waves (Fig 9(b)). This phenomenon is also demonstrated in Figs 5 and 7. In order to resolve this issue, the travelling wave path must be considered. Thus, the calculation of possible propagation paths can be performed as,

$$L_{a}(x_{p},y_{q})=\;∥\varphi_{n}-B_{i_{min},n}∥+\sum_{a=i_{min}}^{(N-\Delta i)/2}∥B_{i_{min},n}-B_{i_{min}+a,n}∥+\sqrt{\varepsilon_{eff,i}}∥B_{i_{min}+a,n}-p∥$$ (14)

$$L_{b}(x_{p},y_{q})=\;∥\varphi_{n}-B_{i_{max},n}∥+\sum_{b=i_{max}}^{(N-\Delta i)/2}∥B_{i_{max},n}-B_{i_{max}-a,n}∥+\sqrt{\varepsilon_{eff,i}}∥B_{i_{max}-a,n}-p∥$$ (15)

Here, ***L***_***a***_ and ***L***_***b***_ are the possible paths with travelling waves in the clockwise and counter-clockwise way from the perspective of antenna and Δ*i* = *i*_*max*_−*i*_*min*_. The optimal paths for each point inside the head is calculated by

$$L_{min}\;\left(x_{p},\;y_{q}\right)=argmin\left[L\left(x_{p},\;y_{q}\right),L_{a}\left(x_{p},\;y_{q}\right),L_{b}\left(x_{p},\;y_{q}\right)\right]$$ (16)

Finally, the total time, ***τ***_*total*_(*x*_*p*_, *y*_*q*_) is calculated from Eq (12) for all (*x*_*p*_, *y*_*q*_) points of H region.

In order to reconstruct the image of head interior a delay-and-sum (DAS) based back-projection technique is utilized. According to DAS, it is assumed that the differential scattered signals, ***D***(*φ*_*n*_, *t*_*k*_) are originated from a suspected point p ϵ H and a coherent summation of the signals originating from surrounding antenna is computed.

$$Image\left(x_{p},\;y_{q}\right)=\left|\sum_{n=1}^{N}\overset{}{\underset{}{\int_{0}^{\infty }\tau_{total}\left(x_{p},\;y_{q}\right)D\left(\varphi_{n},t_{k}\right)dt_{k}}}\right|$$ (17)

The resultant coherent summation for all the antenna responses is large, in case of correct hypothetical scattering point. Contrariwise, if the assumption is incorrect, small value is resulted which can be classified as noise. A continuous colour image is produced by plotting the computed ***Image***(*x*_*p*_, *y*_*q*_) function for each point on the segmented 2D cross-sectional scanned plan of the head phantom and the values are normalized with respect to the maximum of all resulted absolute values. High intensity of colours indicates positions of suspected significant scatterers (haemorrhage) inside the head.

## Results

### Microwave imaging results

To validate the effectiveness of the proposed microwave imaging platform and the reconstruction algorithm, an anatomically realistic 3D human head phantom was imaged. The phantom exterior and various moulds for the different brain tissues were 3D printed. Tissue emulating materials with broadband frequency dispersive dielectric properties from 0.5–4 GHz are filled inside the phantom and skin mimicking tissue materials are also used. The details of the semi-solid head phantom fabrication and properties are mentioned in [51, 52]. To emulate the ICH scenario, hemorrhagic targets with frequency dispersive electrical properties are placed inside the head phantom. The compact microwave transceiver is utilized to collect and store the wideband data from 1.1–3.2 GHz through the antennas in different angular positions around the head. The stored data is then post-processed and the images are reconstructed by using the aforementioned image processing technique. As one of the objectives of the imaging system is to realize its capacity in detecting and locating ICH targets of different sizes and positions, multiple evaluations are performed as described below.

#### A. Imaging of big targets in different locations

**Fig 10:**
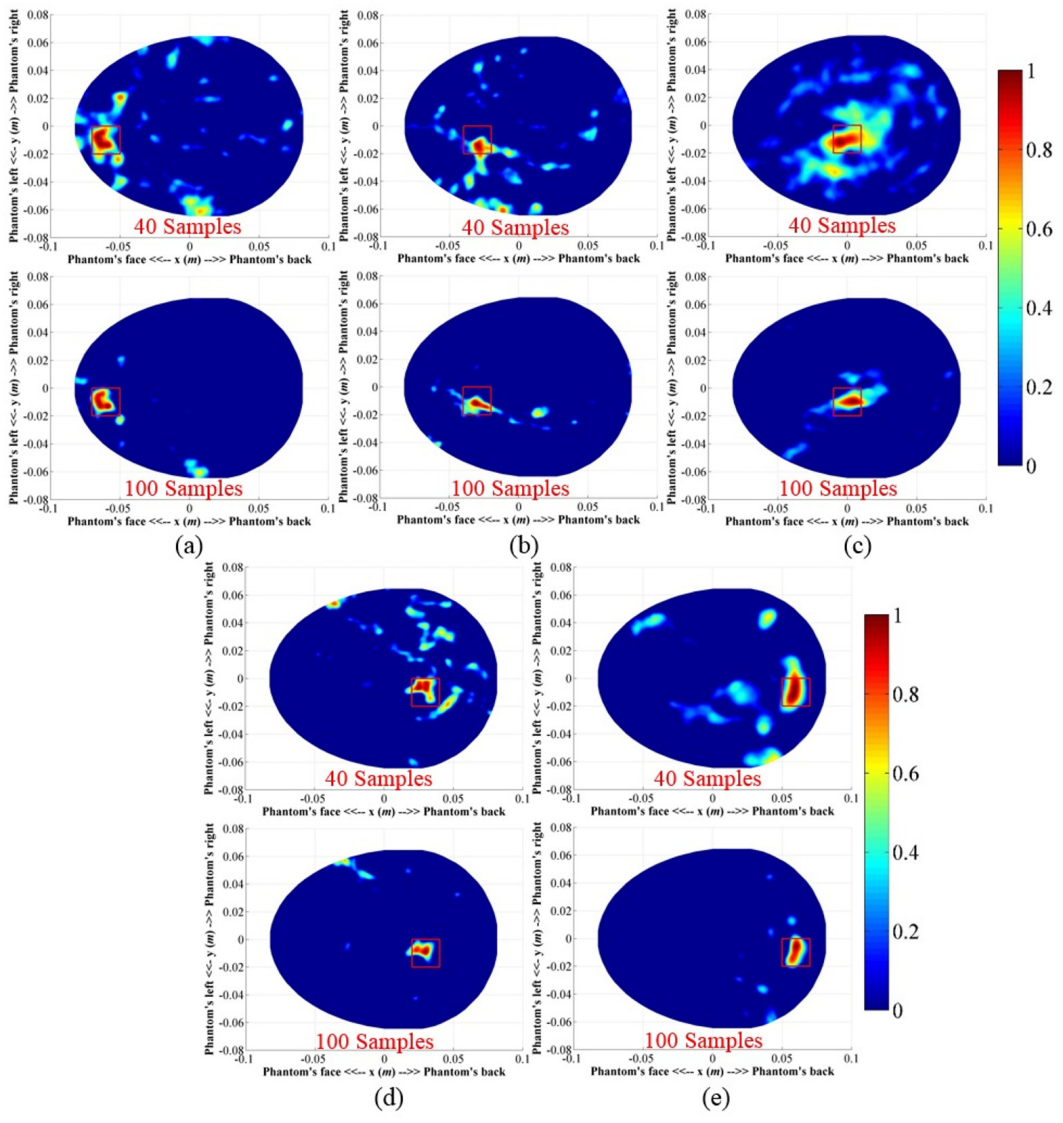
Reconstructed images from the ICH affected head phantom with big targets in different locations and with different data samples (*N* = 40 and 100).

A hemorrhagic target with a volume of 2×2×1 cm^3^ was inserted into the head phantom. Position of the ICH target was varied in depths from the frontal area. Data were collected around the head phantom in two different angular samples, namely N = 40 and 100. The reconstructed images utilizing the imaging algorithm is demonstrated in Fig 10. It is seen that in both cases and for different locations the system is able to detect and localize the positions of the targets. A qualitative assessment of the images indicates that when the number of angular samples is N = 40, more artefacts are visible in all positions. The increased data samples from N = 40 to N = 100 clearly provides better localization.

#### B. Imaging of small targets in different locations

**Fig 11:**
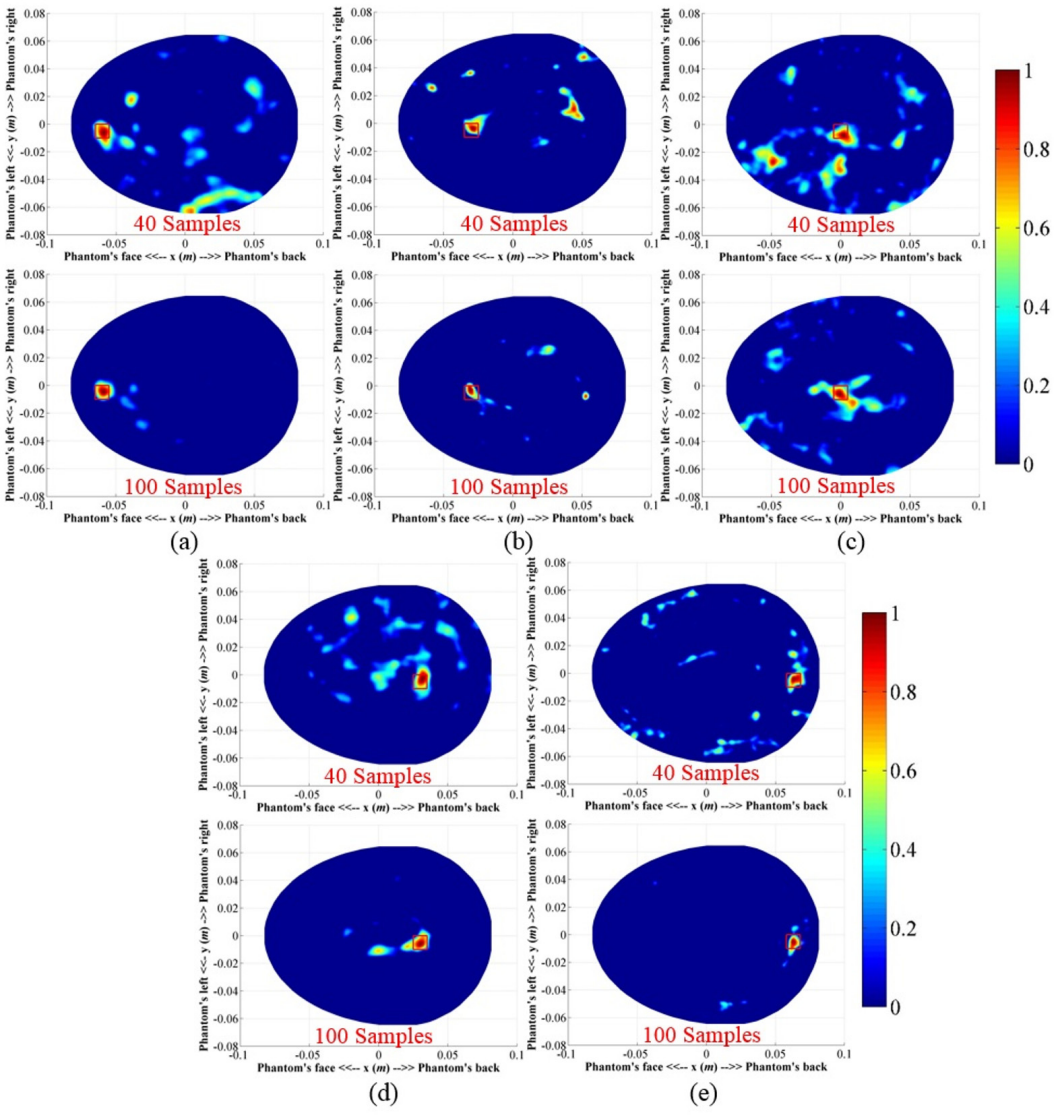
Reconstructed images from the ICH affected head phantom with small targets in different locations and with different data samples (*N* = 40 and 100).

A smaller hemorrhagic target of 1×1×1 cm^3^ is placed inside the head phantom. The experiments are performed for both N = 40 and 100 angular samples. Similar to the previous section, the positions of the targets are varied from shallow to deep locations from the front to back side of the head phantom. The reconstructed images are shown in Fig 11. In both sample cases and different locations, the system is able to detect the strongest scattering inside the red square, which indicates the localization capability of the system. However, the visual inspection elicits that with N = 40, the resulted images contain strong artefacts compared to those of N = 100, suggesting that using a small number of antennas is not efficient in detecting small targets.

### Quantitative analysis methods

In order to investigate the effectiveness of the compact head imaging system and proposed algorithm, several quantitative metrics are investigated by analysing the reconstructed qualities of the reconstructed images [28].

Since a reconstructed image plots significant scatterers with high signal intensities, target recognition from the image can be quantified by the average signal to clutter (S/C) ratio function, Q, which is defined as the relative magnitude of the average intensity values of the points inside the actual target region, T to the rest of the head area, H.

$$Q=10log\left(\frac{\bar{I\left(p\right)}}{\bar{I\left(q\right)}}\right)\;\;\;\left[dB\right]\;\;\;\;\begin{array}{c} \forall p\;\in \;T\\ \forall q\;\in H\;\&\;q\;\notin \;T \end{array}$$ (18)

High value of *Q* indicates high concentration of scattering intensities in actual target region suggesting that the ICH can be identified easily from the reconstructed image. Nevertheless, the correct localization of the target also depends on the recorded maximum intensities of the image inside and outside of ICH target position.

The maximum signal to clutter (S/C) function, *M* is defined by contrast of the maximum intensities of all the points inside and outside the target location, but within the head area. If the whole head and target regions are respectively defined as H and T,

$$M=\frac{max\left[I\left(p\right)\right]}{max\left[I\left(q\right)\right]}\;\;\;\;\;\;\;\begin{array}{c} \forall p\;\in \;T\\ \forall q\;\in H\;\&\;q\;\notin \;T \end{array}$$ (19)

Existence of any strong artefact in the reconstructed image is quantified by the value of *M*. If *M*<1, the position of maximum intensity is outside the actual region (incorrect localization) and the cases where *M*>1 the highest peak is inside the actual target area (correct localization). The value of *M* closer to unity indicates how revealing another artefact is compared to the actual target.

The accuracy of target localization in the resultant image can be described by the parameter, *δ* which is defined as the distance between the true central location of the target, ***c*** and the position of the estimated maximum intensity on the reconstructed image. This accuracy indicator is denoted as,

δ=‖pm−c‖ where pm=arg max[I(p)]      ∀p ∈ H (20)

In ideal case, *δ* has to be zero, but due to the heterogeneous structure and frequency dispersive properties of the actual head tissues, this value is hard to attain.

### Quantitative analysis results

**Fig 12:**
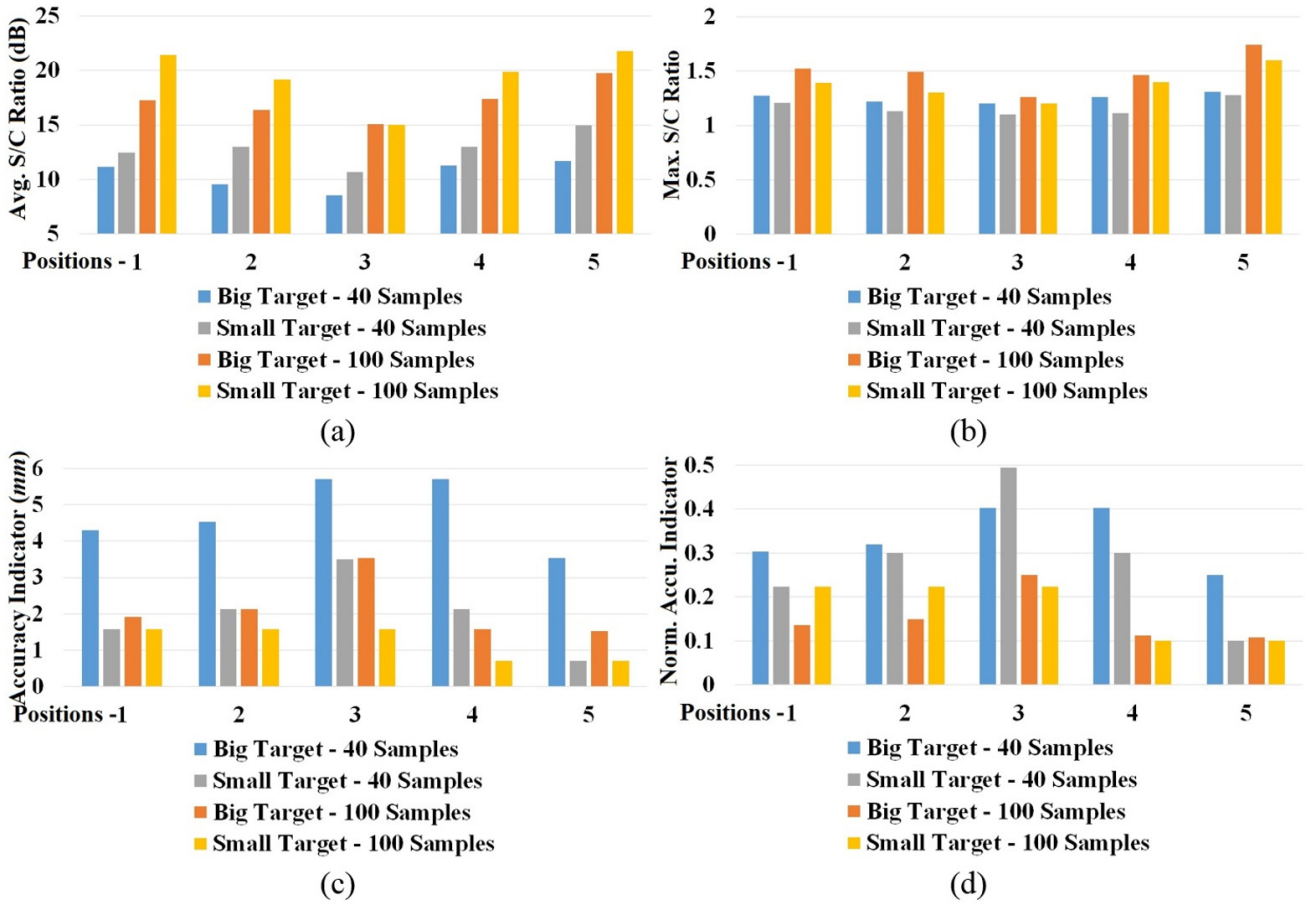
Various quantitative analysis results of the reconstructed images with 40 and 100 data samples and at different positions. (a) Average S/C ratio, *Q*,(b) maximum S/C ratio, *M*, (c) Accuracy indicator, *δ* and (d) normalized accuracy indicator, *δ*_*n*_ are calculated by Eq (18) to Eq (21) accordingly.

A quantitative analysis of the reconstructed images based on the aforementioned metrics was carried out. Fig 12 shows the results of the analysis. High values (>8 dB) of average S/C ratio, *Q* was attained in all cases, which represent that on average the actual target region contains stronger scattering points than the rest of the head cross section. This clearly helps in determining the position of suspected target in practice. The maximum S/C ratio, *M* is also important to distinctly identify the target location from other strong artefacts of the background. It is seen that all the images achieved *M* >1 inside the actual target region, indicating that the reconstruction algorithm is capable to attain correct localization. Fig 12(c) shows that the accuracy indicator is smaller than the distances from the center to the edges of the targets. This infers the detection of maximum intensity inside the actual target region. Since the target sizes are different, the values are normalized to the maximum deviation from the center, *δ*_*m*_ which is half of the diagonal distance from corner-to-corner using the following equation:

$$\delta_{n}=\frac{\delta }{\delta_{m}},\;\;\;\;\delta_{m}=\frac{1}{2}\sqrt{a_{T}{}^{2}+b_{T}{}^{2}}$$ (21)

Here, *a*_*T*_ and *b*_*T*_ are cross sectional dimensions of the target. The results of normalized accuracy indicator are illustrated in Fig 12(d). It is noted that although in actual values it seems that accuracy of big target is much worse than the small target, the insight provided by *δ*_*n*_ reveals that the values are in fact comparable to each other when the sizes of the targets are considered. For both targets, high data samples provide better localization accuracy compared to those of the lower ones.

The results show that the shallow targets provide higher S/C ratios with low accuracy deviation, while targets in the middle (position-3) yield worse metrics in all aspects. Without exception, the values gradually decrease from both front (position-1) and back (position-5) ends. This can be correlated to the quality of the scattered signals received by the antenna. As the target goes deeper into the head phantom, the scattered signals from the target have to pass through more lossy tissue. Consequently, the received scattered signals at the position of the antennas get distorted and attenuated with heavy losses. As a result, the image quality deteriorates with the increase of depth of the ICH target.

To quantify the effect of increasing data sample sizes and detection capability of smaller ICH target sizes, a statistical study was completed. The variations of relative image quality of the head with big and small ICH targets considering both cases when *N* = 40 and 100, are calculated using the equations:

$$Q_{r1}=\frac{\left(Q_{small}-Q_{big}\right)}{Q_{small}},\;\;\;M_{r1}=\frac{\left(M_{small}-M_{big}\right)}{M_{small}},\;\;\;\;\delta_{n}{}_{{}_{r1}}=\frac{\left(\delta_{n}{}_{{}_{small}}-\delta_{n}{}_{{}_{big}}\right)}{\delta_{n}{}_{{}_{small}}}$$ (22)

Again, the relative image qualities for increasing data samples for both big and small ICH targets are computed by:

$$Q_{r2}=\frac{\left(Q_{100}-Q_{40}\right)}{Q_{100}},\;\;\;M_{r2}=\frac{\left(M_{100}-M_{40}\right)}{M_{100}},\;\;\;\delta_{n}{}_{{}_{r2}}=\frac{\left(\delta_{n}{}_{{}_{100}}-\delta_{n}{}_{{}_{40}}\right)}{\delta_{n}{}_{{}_{100}}}$$ (23)

**Fig 13:**
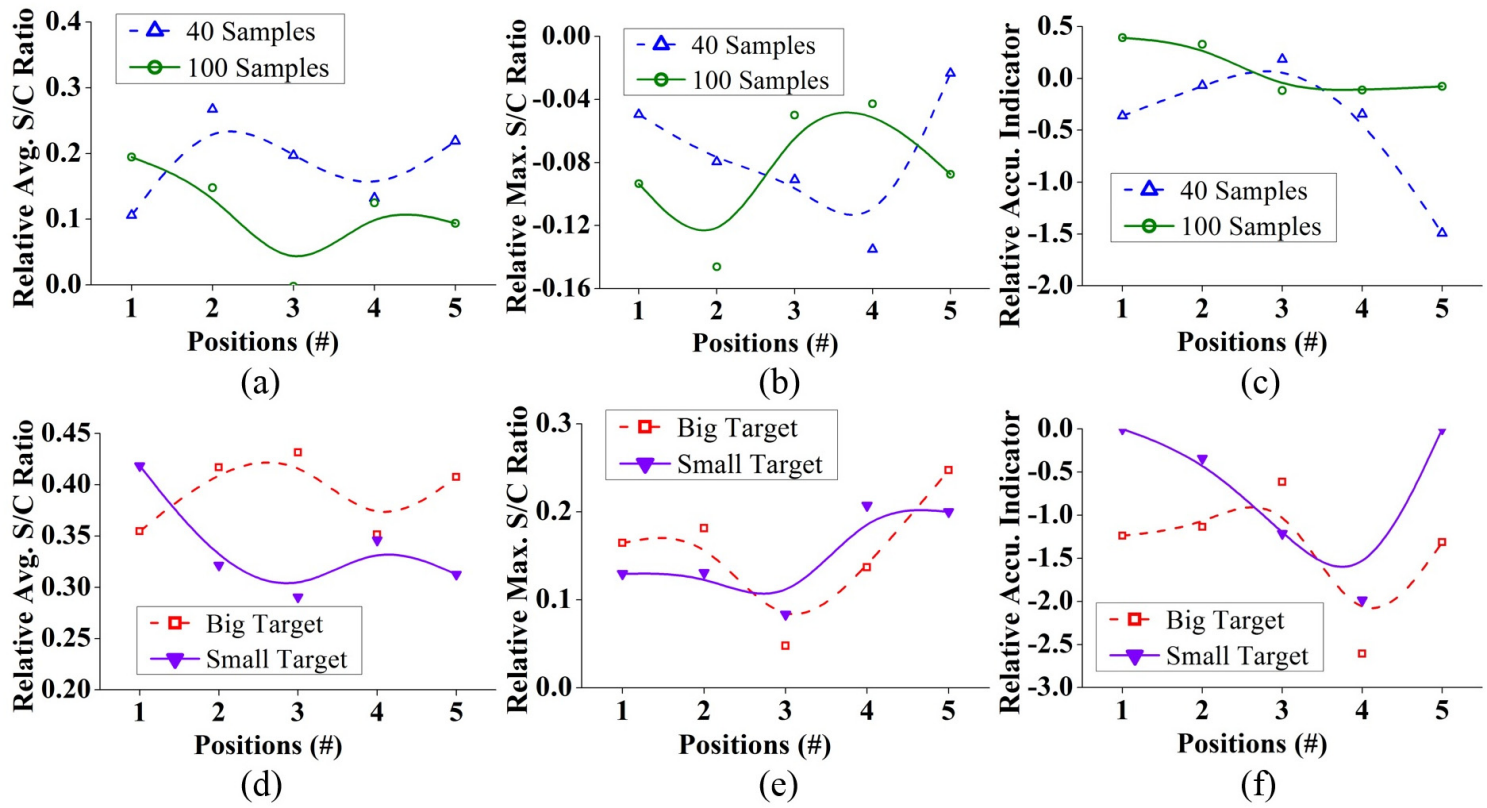
Statistical analysis results of the quantitative metrics obtained from the reconstructed images calculated from Eqs (22) and (23) for different samples and targets.

Results from Eqs (15) and (16) are shown in Fig 13. The relative *Q*_r1_ values for both 40 and 100 samples (Fig 13(a)) show positive variations indicating an improvement of average image quality. Due to the considered smaller cross-section of the small target with the same head phantom, an increase of average S/C ratio, *Q*, is expected. Negative relative *M*_r1_ values (Fig 13(b)) indicate that although the targets are properly detected, the scattering of the small target is weaker than the big target. This also suggests that more artefacts are present in the reconstructed images for small ICH target. This is because scattering signals from other parts of head tissues are relatively larger in the sensing antenna than the scattering from a small target. Positive and negative fluctuations of relative accuracy indicator, ${\delta_{n}}_{r1}$ (Fig 13(c)) conveys that no conclusive improvement or decline of target accuracy detection can be obtained when reconstructed images from big and small targets are compared. The reason behind this lies in the physics of the target’s inherent scattering characteristics. A close examination of Figs 5, 6, and 7 reveals that the maximum intensity of the target’s scattering is not a fixed point. Although the point with maximum scattering field exists within the target region, its position vary over the operating frequency band as well as with the position of the ICH target. Nevertheless, changing the excitation source position alters the maximum scattering point for the same target and functional frequency. For these reasons, the position of maximum intensity point in the reconstructed images vary unpredictably.

When the data samples are increased from *N* = 40 to 100 in both big and small target cases, the relative average (*Q*_r2_) (Fig 12(d)) and maximum (*M*_r2_) (Fig 12(e)) S/C ratios show positive variations, whereas the relative accuracy indicator (${\delta_{n}}_{r2}$) (Fig 12(f)) exhibits negative variations. Hence, it can be concluded from this study that increasing data samples from *N* = 40 to 100 enhances the reconstructed images for all targets sizes and at all locations.

The relative metrics are further analysed to quantify the amount of dispersion of the matrix values. Table 2 lists the percentage standard deviations and means of the relative metrics in different scenarios calculated with a 99% confidence level. The results indicate a mean improvement of more than 11% in *Q*_r1_ and reduction of around 8% in *M*_r1_ with percentage standard deviation (SD) of less than 7%, which represents low variations from the mean deviations. Rapid fluctuations of accuracy indicator (${\delta_{n}}_{r1}$) yields negative (-41.62%) and positive (8.29%) mean values respectively for N = 40 and 100 with a huge SD, as high as approximately 58%, demonstrates the non-monotonous variations of maximum intensity points. The points of maximum intensity in the reconstructed images are always found inside the actual target region (Figs 10 and 11), showing the efficacy of the system for both big and small targets.

**Table 2:** Standard deviation and mean analysis of the relative quantitative metrics.

	ICH size variations		Data sampling variations	
***Metrics***	***Q***_***r*1, *N* = 40**_	***Q***_***r*1, *N* = 100**_	***Q***_***r*2, *Big***_	***Q***_***r*2, *Small***_
**SD (%)**	5.86	6.57	3.31	4.41
**Mean (%)**	18.43	11.17	39.24	33.78
***Metrics***	***M***_***r*1, *N* = 40**_	***M***_***r*1, *N* = 100**_	***M***_***r*2, *Big***_	***M***_***r*2, *Small***_
**SD (%)**	3.79	3.69	6.50	4.69
**Mean (%)**	-7.57	-8.4	15.55	15.02
***Metrics***	${\delta_{n}}_{r1,\;N\;=\;40}$	${\delta_{n}}_{r1,\;N\;=\;100}$	${\delta_{n}}_{r2,\;Big}$	${\delta_{n}}_{r2,\;Small}$
**SD (%)**	57.46	22.82	65.97	77.81
**Mean (%)**	-41.62	8.29	-138.3	-70.86

On the other hand, increasing data samples from N = 40 to 100 results more than 33% and 15% improvements in *Q*_r2_ and *M*_r2_, respectively, and more than 70% reduction of accuracy error. Although small SD of around 4% for *Q*_r2_ and as low as around 5% for *M*_r2_ are resulted from the statistical analysis, in case of ${\delta_{n}}_{r2}$ a high SD of around 70% is observed. The improvements of these relative mean values are higher and SD values are mostly lower in case of the big target when compared with the small target. This is because, for the relatively stronger scattering fields from the big target, the data sample increment gives better reconstructed images.

## Discussion

A mono-static head imaging system relying on the wideband microwave frequencies 1.1–3.2 GHz has been presented in this manuscript. The system utilizes a custom-designed ultra-wideband antenna as sensing element to scan the head. It is observed that the half-cut technique effectively reduces the space requirement for the sensing element, whereas the modified cross-feeding technique corrects the main beam-tilting issue. As a result, the proposed antenna is capable of radiating efficiently along the front direction. In the forward direction. Nevertheless, the compact size of the antenna confines the phase center radiation to a stable position over a wide bandwidth, which can be quantified by 98% fidelity factors [28]. These radiation features improve the detection capability of the image processing algorithm because of the basic assumption that all radiation starts from a single point, which is difficult to realize in other antennas [53, 54].

The numerical analysis of the penetration of transient signals and the scattering of ICH targets of different sizes in diverse locations inside MRI-derived realistic head model reveals the operating mechanism of the head imaging system. In a human head model with above average size, the transient E-field signals attenuate by around 45 dB (Fig 4) at the center when travelling through the longest radius (~100 *mm*). A high level of attenuation is therefore expected for deep targets. Owing to the stable phase center of the antenna, the level of impulse distortion, which is quantified by fidelity factors, is found to be high (~ 80%) at the center. On the other hand, the scattering characteristics of ICH targets in the frequency domain demonstrates that the lower frequencies are less susceptible to the losses (Fig 5) compared to the higher microwave frequencies. Shallow haemorrhages are seen to scatter the co-polarized E_z_-fields strongly relative to the deep one. However, when the antenna is facing the small radius rather than the longest, it is noted that scattering signals even at high frequencies from deep target become comparatively stronger (Fig 6) owing to reduced losses in this orientation. This indicates that scanning from various perspectives is vital for adequate data acquisition, especially for the deep targets. Nevertheless, it is demonstrated in Fig 7 that the maximum co-polarized scattering E_z_-fields from an ICH target are contingent on target’s size (Fig 7). Depending on the position of the target, a reduction of 8–10 dB in the maximum scattered field strength is observed when a small target (1×1×1 cm^3^) is compared to a large one (2×2×1 cm^3^).

A modified delay-and-summation back-projection algorithm is proposed for the signal and image post-processing from the head imaging system. The data from the imaging system is acquired in the frequency-domain and converted to the time-domain for post-processing. Data acquisition in the frequency-domain is preferred because of the beneficial high dynamic range of frequency-domain measurements [55]. As it is noted from Fig 5, even though the target (target-3) is away from the antenna, it can receive signal and scatter back through surface waves. Hence, the proposed imaging algorithm includes the effect of surface waves while calculating the minimum possible paths from a specific point inside the head model to the sensing antenna. Instead of using a conventional single constant *priori* of head permittivity, distance dependent effective permittivity model which is contingent on the point of entry inside the head model is utilized.

The efficacy of the system is verified by imaging ICH targets of different sizes and in different positions inside a realistic human head phantom. The reconstructed images demonstrate that the imaging system can successfully detect and localize ICH. The quantitative analyses illustrate that when the target gets deeper inside the head, the quality of images in terms of average and maximum signal-to-clutter ratio (S/C) and accuracy indicator deteriorate for targets of any size due to relatively lower scattering signals as observed in the numerical studies (Fig 5). Again, when compared between targets of different sizes located at the same positions, it is noted that the maximum S/C decreases for small targets. This result indicates that as the targets become smaller, the scattering from other parts of the brain become relatively stronger than the actual ICH scatterer. However, literatures on ICH patients report that at early stages, the median volume of ICH targets is 17 cm^3^ [56] or generally in the scale of centimetres [57]. Thus, the experimental detection of both big (2×2×1 cm^3^) and small (1×1×1 cm^3^) targets can be considered as early goals.

The relative statistical analysis illustrates that the image quality can be significantly improved and the presence of artefacts can be debased by increasing the number of data samples (*N*) collected from around the head phantom. Statistics shows that irrespective of the size of ICH targets, when the number of samples (*N*) are increased from 40 to 100, the average and maximum S/C are enhanced by more than 33% and 15%, respectively with SD of less than 7% for both metrics with an improvement of more than 70% in the accuracy indicator. The statistical analyses were performed with a 99% confidence level. As the image quality declines, in case of small ICH targets if non-definitive results are obtained (which can even happen in case of CT [58]) and it is only the proposed head imaging system that can be easily adjusted to produce images with higher quality while only a single antenna calibration is required. On the other hand, an adjustable array-based counterpart [26] can also be rotated using a stepper motor, it requires antenna array calibrations and can waste precious time delaying the medication of the patient. However, since the proposed head imaging system operates over a wide bandwidth, it is affected by noise. By utilizing Gaussian white noise on the measured signals [29], it is found that for such a microwave imaging system the signals to noise ratio needs to be more than 10 dB in order to attain a distinguishable target detection overcoming the noise floor. Such signal to noise ratio is usually easy to attain in practical scenarios.

As the head shapes and sizes vary from one patient to another and in MI system sensing antennas should be placed as close as possible, the proposed single antenna system can be quickly adjusted compared to the array based system. Owing to the light weight and compact size of the proposed mono-static scanning system, it is flexible and faster to move around the head phantom in a circular scanning profile, while bulky arrays takes more space for the setup and more time to move because they are heavier.

The proposed system overcomes the strong multi-path reflection problem from the other antennas [19, 20]. Although utilization of a single-antenna system is virtually equivalent to the array based system, the scattering contribution of the neighbouring elements might be significant in array-based systems due to the presence of surface waves around the head, even if all the remaining array elements except the active antenna are match terminated [21]. This added scattering in the array-based system increases the presence of artefacts, while the proposed single antenna head-imaging system is free from such artefacts in the reconstructed images. Moreover, when compared to the previously reported systems, the presented MI system is able to detect the location of targets as small as 1×1 cm^2^ of cross-section in both deep and shallow locations, which was not possible in the reported systems [28, 29].

**Fig 14:**
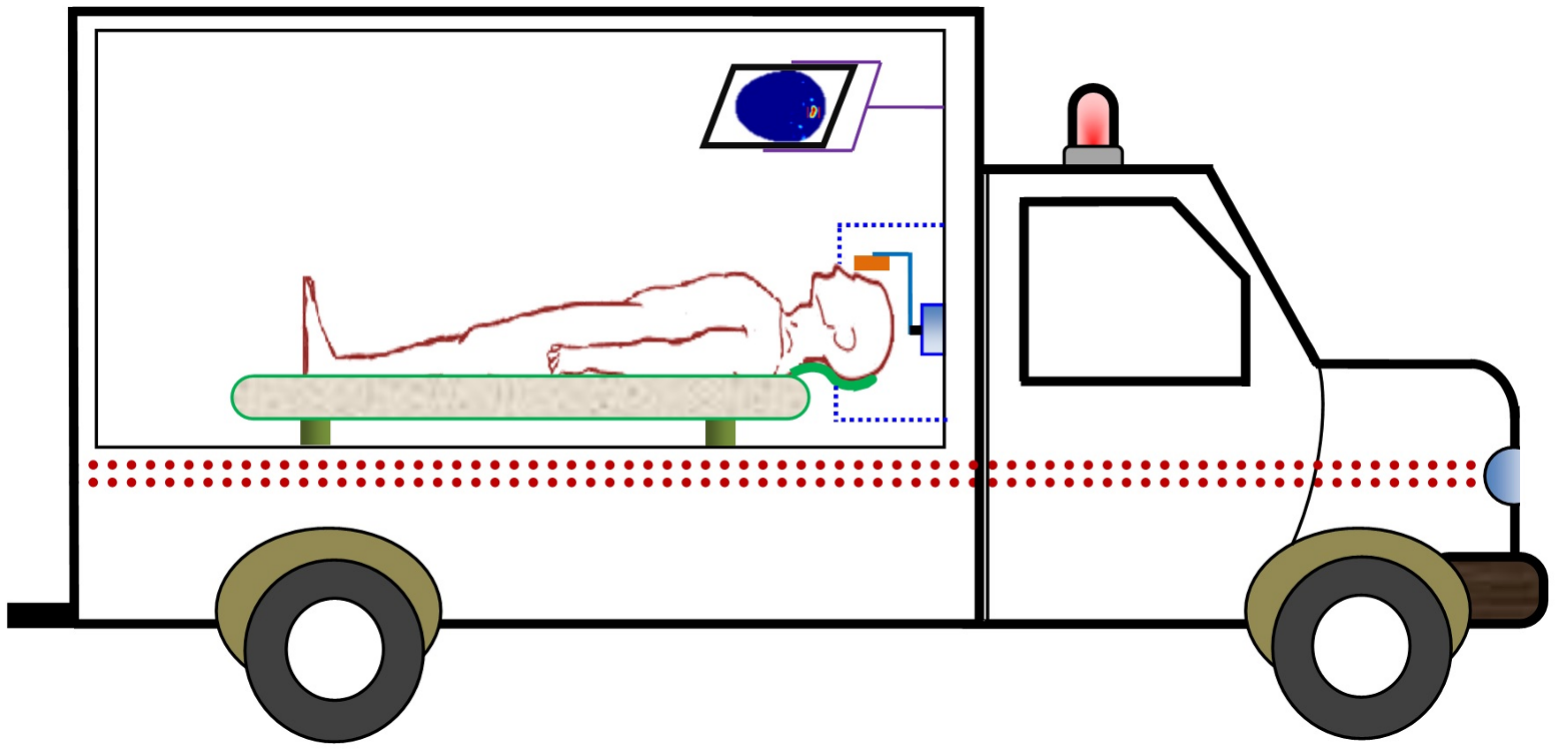
Schematic illustration of the proposed head imaging system in an ambulance.

The prototyped head imaging system is compact and light weight and could be carried in an ambulance. The system does not require large computing resources, with the assistance of the fast and accurate image post-processing, reconstruction can be performed in standard portable devices. An illustration of how the imaging system could be applied in an ambulance is illustrated in Fig 14. A support made from microwave frequency transparent materials can be used to support patient’s head. The data acquisition time is around 1 minute for *N* = 100 data samples, and it only takes 10–12 seconds for the algorithm to generate the resultant image. Future work is planned to perform pilot human testing on healthy volunteers once safety and ethics approvals are in place.
